# Approaches to characterising multimorbidity in older people accessing hospital care: a scoping review

**DOI:** 10.1007/s41999-025-01166-3

**Published:** 2025-03-01

**Authors:** Jonathan G. Bunn, Lewis Steell, Susan J. Hillman, Miles D. Witham, Avan A. Sayer, Rachel Cooper, the ADMISSION research collaborative

**Affiliations:** 1 AGE Research Group, Translational and Clinical Research Institute, Faculty of Medical Sciences, Newcastle University, Newcastle upon Tyne, UK; 2 NIHR Newcastle Biomedical Research Centre, Newcastle upon Tyne Hospitals NHS Foundation Trust, Cumbria Northumberland Tyne and Wear NHS Foundation Trust and Faculty of Medical Sciences, Newcastle University, Newcastle upon Tyne, UK

**Keywords:** Multimorbidity, Complexity, Older adults, Hospital

## Abstract

**Purpose:**

An increasing proportion of older adults accessing hospital care are living with multimorbidity, with a high degree of complexity of multimorbidity in older hospital populations expected. We aimed to assess approaches taken to characterise multimorbidity in older adults accessing hospital care, including how complexity is considered.

**Methods:**

Following established scoping review guidelines, all published studies that characterised multimorbidity in a hospital population, with average age ≥ 65 years, were identified via a prespecified search strategy. Six electronic databases were searched to identify peer-reviewed literature published to September 2023 meeting eligibility criteria. Screening was undertaken by two independent reviewers, and data extracted using a standard proforma.

**Results:**

Of 5305 titles and abstracts screened, 75 papers, reporting on 72 unique study populations across 24 countries, met inclusion criteria. There was heterogeneity in most aspects of characterisation. Multimorbidity was defined in 43% (*n* = 31/72) of studies; most (*n* = 59/72, 82%) aimed to describe a multimorbidity-outcome association. Number of conditions considered ranged from 2 to 285 and weighted indices were used as a measure of multimorbidity in 75% (*n* = 54/72) of studies, with 56% (*n* = 40/72) using a version of the Charlson Comorbidity Index. Complexity was explicitly studied in 17% (*n* = 12/72) of studies.

**Discussion:**

Our review highlights heterogeneity in characterisation of multimorbidity in older adults accessing hospital care, with limited consideration of complexity. As the proportion of older adults accessing hospital care who are living with multimorbidity increases, better characterisation of their multiple conditions and associated complexity is a priority to ensure delivery of appropriately tailored care.

**Supplementary Information:**

The online version contains supplementary material available at 10.1007/s41999-025-01166-3.

## Key summary points

**Aim:**

To assess approaches taken to characterise multimorbidity in older adults accessing hospital care, including an assessment of how clinical complexity has been accounted for.

**Findings:**

This review identified 75 papers reporting on 72 unique study populations. Only 43% (*n* = 31/72) of these studies of multimorbidity in older adults accessing hospital care explicitly defined multimorbidity, and there was a reliance on weighted indices (61%, *n* = 41/72) to characterise multimorbidity. Complexity was rarely explicitly studied (17%, *n* = 12/72), and where it was no consistent approach to exploring complexity of conditions or health and care interactions within this population was evident.

**Message:**

As the proportion of older adults accessing hospital care who are living with multimorbidity increases, better characterisation of their multiple conditions and associated complexity is a priority to ensure delivery of appropriately tailored care.

**Supplementary Information:**

The online version contains supplementary material available at 10.1007/s41999-025-01166-3.

## Background

Multimorbidity (also termed multiple long-term conditions, MLTC) is the coexistence of two or more long-term physical, mental health, or infectious conditions in an individual [1]. Multimorbidity is a global challenge for healthcare systems and its impacts are forecast to increase as prevalence rises in the coming decades [1–3]. Research on multimorbidity has been hampered by lack of consensus on its definition and operationalisation. However, recent attempts to highlight this and bring about consensus have begun to provide clarity and lead to greater transparency in reporting and consistency within the field [4–7].

Age is one of the primary risk factors for the development of multimorbidity, with the proportion of people aged over 60 years living with multimorbidity globally reported as greater than 50% [8]. Prognosis for older adults living with multimorbidity is significantly worse than for older adults with no or single conditions, particularly when a mental health condition is present [9]. Higher symptom burden, increased risk of functional decline and reduced quality of life, are all associated with multimorbidity, so there is a need to improve our understanding of multimorbidity with the aim of mitigating these adverse outcomes [10–12]. A key challenge for older adults living with multimorbidity is transitions of care, with management of multiple conditions involving interactions with a range of different health and care services [13]. However, the characteristics of people living with multimorbidity who are interacting with hospital services has received limited attention, and to improve outcomes for older adults living with multimorbidity this needs to be addressed.

A systematic review by Ho et al. [4] examined variation in the measurement of multimorbidity across 566 studies published to January 2020 that had measured multimorbidity for any purpose in any setting. They found that measurement of multimorbidity was poorly reported and highly variable and urged researchers in the field to report the definition of multimorbidity used within their work. In this systematic review, only 18.4% of included studies described multimorbidity in a hospital population. Whilst the conclusions and recommendations identified by the Ho et al. systematic review are informative, the discrete nature of hospital populations means more granular knowledge of how multimorbidity has been characterised in this population would be beneficial [14]. People with multimorbidity accessing hospital care represent a distinct subset of individuals living with multimorbidity, whereby their acuity of disease is enough to require interaction with hospital services and thus the patterns, aetiology, and prognosis of multimorbidity may differ [14]. Studies have shown that multimorbidity is associated with higher admission rates, prolonged length of stay and higher rates of inpatient mortality, but the broader impacts of multimorbidity on the individual, such as quality of life, have received less attention [15]. A review of qualitative studies has highlighted that individuals living with multimorbidity who access hospital care often report poor service integration and a lack of person-centred care, with healthcare professionals within the hospital often lacking agency and capacity to manage conditions outside their specialty [13, 16].

Older people account for the majority of hospital attendances and admissions, and with multimorbidity predicted to rise within the population aged over 65, the complexity of these hospital interactions is likely to increase [17]. Data published in 2024 have shown that the proportion of people admitted to hospital who have multimorbidity is rising substantially; for example, the proportion of people aged 65 years and older admitted to hospital in England with 3 or more long-term conditions increased from 19.8 to 55.0% between 2006 and 2021 [18]. The challenge of developing a strategy to meet the requirements of this population, is the proportion and heterogeneity of people who are identified as living with multimorbidity using the standard ‘two or more conditions’ approach. For example, in one study of 1,751,841 individuals in Scotland receiving primary care, over 80% of over 85 s were reported to be living with multimorbidity [19]. A target subpopulation likely exist who would benefit from tailored management of their multimorbidity. As described by Yarnall et al. [20], this target population likely have a combination of severity of individual conditions and complexity of care, such that a tailored management approach of multimorbidity is required. Through understanding how multimorbidity has been characterised in older adults (aged ≥ 65 years old) accessing hospital care, we aim to identify how the challenge of characterising this population and its complexities of health and care has been achieved to date. We, therefore, undertook a scoping review, aiming to:

1. Identify approaches that have been taken to characterise multimorbidity in older people accessing hospital care, and the extent to which approaches take account of the complexity of conditions and care.
2. Summarise outcomes that have been studied in relation to older people with multimorbidity who access hospital care.
3. Describe key gaps in the literature that need to be addressed to further our understanding of multimorbidity in older adults accessing hospital care.

## Methods

The scoping review framework proposed by Arksey and O’Malley [21], further developed by Levac et al. [22], and Peters et al. [23], was used to establish our process. This included: identifying the research question; identifying relevant studies; study selection; data charting; collating, summarising, and reporting results. We were also guided by the Preferred Reporting Items for Systematic Reviews and Meta-Analyses extension for Scoping Review (PRISMA-ScR) checklist (see Supplementary Information 1) [24]. Using the scoping review framework and the PRISMA-ScR checklist, we developed a prespecified protocol (see Supplementary Information 2) [21–24].

## Research question and search strategy

The research question was formulated using the CoCoPop (Condition-Context-Population) framework [25]; the condition was multimorbidity, the context hospital populations, and the population adults aged 65 years and older. The search strategy was developed from the systematic review by Ho et al. [4]. Firstly, all studies included within the systematic review by Ho et al. were included for initial screening within our review. Then, using an adapted version of the search strategy conducted by Ho et al., we identified any literature published from the end of their review (21st January 2020) until 7th September 2023. Updated searches were undertaken in the following databases, with support from a medical librarian: CINAHL via EBSCO; Ovid (MEDLINE, Embase and PsycINFO); Scopus; Web of Science; The Cochrane Library; CAB Direct (Global Health). Unlike the Ho et al. review, ProQuest Dissertations and Theses Global databases were not searched due to our decision to exclude grey literature. The search strategies and results for each database are presented in Supplementary Information 3. Study titles and abstracts were extracted into EndNote (version 20), for initial deduplication. Following this, titles and abstracts were uploaded to Rayyan (2022) [26], an online systematic review software package, where automation tools were used to aid any further deduplication.

Screening of titles and abstracts was undertaken against prespecified eligibility criteria by four reviewers (JB, LS, SH, and RC). Studies were included if they were quantitative and described multimorbidity in a hospital setting within a population with an average age ≥ 65 years (for full description of eligibility criteria see Table 1). All titles and abstracts were independently reviewed by two of the four reviewers. Disagreement or uncertainty around inclusion decisions were discussed within the wider authorship group to achieve consensus. The following data were extracted from included studies (by JB) using a standard proforma (see Supplementary Information 4): author(s); year of publication; country of study; demographic data on population size and sex; age (and measure of average used within the study); type of hospital care accessed; data source of condition data (for example medical records); multimorbidity definition; number and name of health conditions; study aims; method of characterising multimorbidity (simple counts or weighted indices); account of complexity; outcomes of interest; key findings. In accordance with methodological guidance for scoping reviews, formal critical appraisal of included studies was not performed during extraction [21, 23].

**Table 1:** Eligibility criteria for study inclusion and exclusion for assessment of titles, abstracts, and full texts

Descriptor	Inclusion criteria	Exclusion criteria
Study type	Quantitative	Qualitative studies, mixed methods studies, study protocols, literature reviews, editorials, and commentaries
Age of participants	Study population all aged ≥ 65 years or average age of the study population is ≥ 65 years	Studies that have no age analysis to determine the age distribution of the population or where the reported average of the study population is < 65 years
Setting	Hospital: emergency department, inpatient, and outpatient secondary or tertiary care, where care is delivered by specialists who are not the individuals’ usual medical practitioner	Community: primary care, community hospitals, virtual wards, or other forms of hospital at home
Definition of multimorbidity	Any paper that examines multimorbidity without focus on a specific condition	Studies of comorbidity i.e. which focus on an index condition in the presence of one or more other conditions
Peer review	Peer-reviewed papers	Studies that have not been peer reviewed, grey literature
Language of publication	English language	Not English
Study date	Studies included in the Ho et al. searches were from inception of the database to 21/1/2020 Replication and extension of Ho et al. searches from 21/1/2020 to 7/9/2023	

### Collating, summarising, and reporting of results

Following initial extraction, studies were summarised using a narrative approach by country of origin, the approach used to define and characterise multimorbidity, and by account of complexity. Complexity does not have a universally accepted definition; we therefore developed a pragmatic approach to describe the variation in approaches. Studies were determined to have explicitly or implicitly accounted for complexity. Explicit approaches were those that specifically described or defined a group with severe or complex multimorbidity or clinical complexity, with those that focussed on this characterisation throughout their work deemed to have featured complexity as a major part of their analyses. The inclusion of three terms (severe multimorbidity, complex multimorbidity and clinical complexity), reflects the use of these terms interchangeably within the literature, and ensured capture of relevant discussion whichever terms were used. Implicit approaches were those that referred to wider concepts of complexity, such as the functional impact of conditions, but did not specifically describe or define a group with severe or complex multimorbidity or clinical complexity.

## Results

### Overall characteristics of eligible studies

**Fig. 1:**
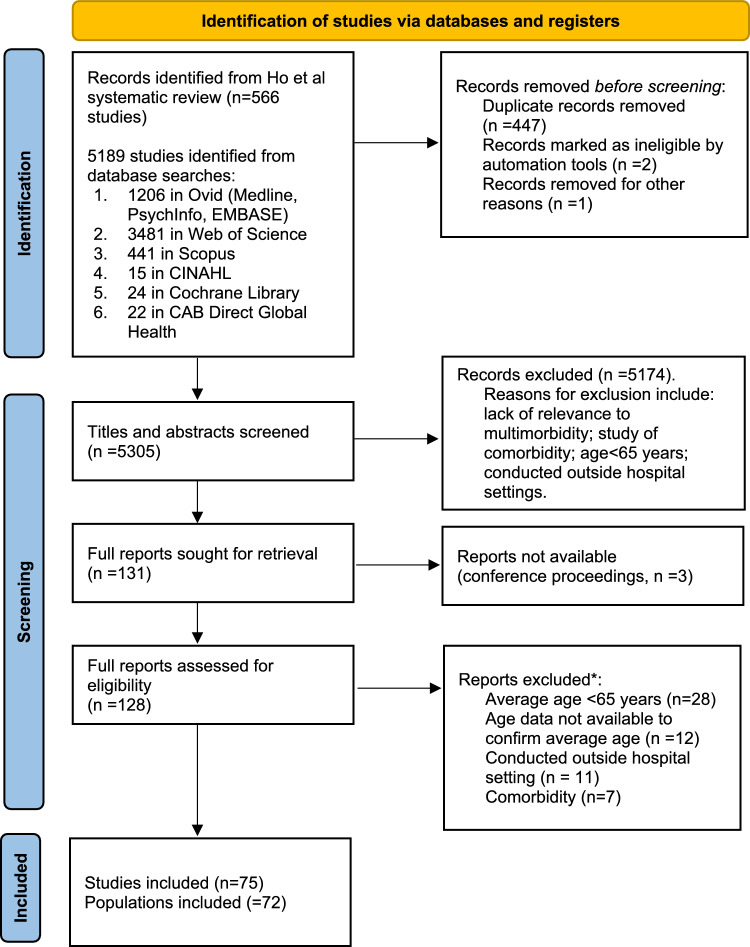
PRISMA flow diagram. *Note that during assessment of full reports 5 were excluded for more than one reason

A total of 5305 records were screened (see Fig. 1), with 75 eligible papers, describing 72 unique study populations, identified (see Table 2) [27–101]. For full extraction details, see Supplementary Information 5. Studies were conducted in 24 countries, with the USA (*n* = 15/72, 21%) and China (*n* = 8/72, 11%) being the most common countries of study (see Supplementary Information 6, Table 1). However, of the remaining studies, the majority were conducted in Europe (*n* = 36/72, 50%): though many European countries were only represented within a single published study. The median size of study population was 618 (range 80–4,199,002), with populations tending to contain more women than men (*n* = 52/72, 72%).

**Table 2:** Characteristics of included studies and approaches for characterising multimorbidity

	Number of populations (*n* = 72)	Reference numbers of studies
*Study setting*		
Inpatient	53 (74%)	[27–29, 32, 34–38, 40, 41, 45, 46, 48–50, 52–54, 56–59, 62–64, 66–84, 86, 87, 90, 91, 94, 95, 97–101]
Outpatient	7 (10%)	[30, 31, 39, 42, 85, 88, 92]
Emergency Department (ED)	6 (8%)	[47, 51, 55, 61, 89, 96]
Outpatient and inpatient	4 (6%)	[43, 60, 65, 93]
ED and inpatient	1 (1%)	[33]
Unknown	1 (1%)	[44]
*Data source*		
Medical records and administrative databases	54 (75%)	[27–29, 33, 35–37, 42, 43, 45–53, 55, 56, 58–60, 62, 63, 65–68, 70–80, 82–88, 91–98, 100, 101]
Self-report	6 (8%)	[30, 31, 44, 57, 69, 100]
Self-report and medical records and administrative databases	7 (10%)	[32, 34, 39, 40, 68, 81, 89]
Not reported	5 (7%)	[38, 41, 61, 64, 90]
*Population size*		
0–99	3 (4%)	[40, 57, 62]
100–999	38 (53%)	[27, 28, 30–32, 34–39, 41, 44, 46, 48, 53–56, 58, 59, 61, 67, 69, 74, 76, 77, 80–83, 89–93, 96–98]
1000–9999	13 (18%)	[42, 45, 47, 49–51, 68, 72, 73, 84, 85, 99–101]
10,000–99,999	7 (10%)	[29, 33, 43, 66, 75, 88, 94, 95]
100,000+	11 (15%)	[52, 60, 63–65, 70, 71, 78, 79, 86, 87]
*Aim(s) of study*		
Association of multimorbidity with an outcome	59 (82%)	[27–29, 32, 34–36, 38, 40, 42, 43, 45, 47, 51, 52, 55, 57–59, 62–67, 69–81, 85–92, 94–98, 100, 101]
Validation of the measure in characterising multimorbidity	16 (22%)	[32, 34, 42, 49, 50, 52, 53, 55, 66, 69, 72, 73, 75, 80, 89, 92, 97, 98]
Study of populations with multimorbidity	15 (21%)	[33, 39, 44, 46, 54, 56, 60, 64, 67, 68, 83–87]
Prevalence or burden of multimorbidity	8 (11%)	[30, 31, 37, 71, 82, 91, 93, 99]
Patterns or groups (clusters) of multimorbidity	4 (6%)	[41, 48, 49, 61]
Risk factors for developing multimorbidity	1 (1%)	[99]
*Definition of multimorbidity*		
Not reported	41 (57%)	[27, 28, 30–32, 34–36, 38, 40, 42, 45, 47, 49–51, 53, 55, 57–59, 62, 63, 65, 67, 69, 70, 74, 76, 77, 80, 81, 86, 87, 89, 90, 92, 94–98, 100]
2 or more conditions	27 (38%)	[29, 37, 39, 41, 43, 44, 46, 48, 52, 54, 56, 60, 61, 64, 66, 68, 71–73, 75, 82, 83, 85, 91, 93, 99, 101]
3 or more conditions	1 (1%)	[84]
Presence of specific groups of conditions^a^	3 (4%)	[78, 79, 88]
*Measure of multimorbidity*		
Simple count of conditions	17 (24%)	[30, 31, 37, 39, 40, 43, 44, 46, 48, 56, 60, 61, 68, 71, 85, 93, 99]
Weighted index	44 (61%)	[27, 28, 32, 33, 35, 36, 38, 41, 42, 45, 47, 49–51, 53, 55, 57–59, 62, 64–66, 70, 72–79, 82–84, 86–90, 92, 94–98, 100, 101]
Both simple count and weighted index	10 (14%)	[29, 34, 52, 54, 63, 69, 80, 81, 86, 87, 91]
Unclear	1 (1%)	[67]
*Number of conditions within measure of multimorbidity*		
Not reported	10 (14%)	[30, 31, 44, 55, 56, 64, 67, 77, 85, 93]
2–15	10 (14%)	[42, 46, 49, 50, 57, 68, 71, 76, 82, 83, 99]
16–30	22 (31%)	[27, 28, 32, 33, 35–37, 39, 40, 45, 47, 51, 54, 58, 61, 74, 89, 90, 94–96, 100, 101]
31–45	2 (3%)	[43, 65]
46–60	1 (1%)	[59]
> 60	5 (7%)	[29, 48, 60, 78, 88]
Not applicable (studies that examined multiple measures)	22 (31%)	[34, 38, 41, 52, 53, 62, 63, 66, 69, 70, 72, 73, 75, 79–81, 84, 86, 87, 91, 92, 97, 98]
*Account of complexity*		
Complexity explicitly accounted for	12 (17%)	[36, 39, 48–50, 64, 66, 72, 73, 82, 83, 85–87]
Complexity implicitly accounted for	22 (31%)	[29, 37, 38, 43, 44, 52, 54, 58, 61–63, 65, 67, 69, 71, 80, 81, 84, 91, 93–95, 97, 98]
Complexity not accounted for	38 (53%)	[27, 28, 30–35, 40–42, 45–47, 51, 53, 55–57, 59, 60, 68, 70, 72–79, 88–90, 92, 96, 99–101]

^a^Studies utilise Qualifying Comorbidity Sets (QCS), which ascribe presence of multimorbidity to the presence of a QCS. QCS are either single, or more commonly, groups of specific conditions

The most common aspect of hospital care studied was inpatient care alone (*n* = 53/72, 74%) followed by outpatient care alone (*n* = 7/72, 10%) and emergency department (ED) care alone (*n* = 6/72, 8%) (see Table 3). Data on conditions and sociodemographic factors were most often drawn from medical records and administrative databases (*n* = 54/72, 75%), with self-reported data less commonly used (*n* = 6/72, 8%) (see Table 2).

**Table 3:** Summary of weighted approaches, and the process by which weighting occurs

Index	Number of study populations (*n* = 72)	Reference numbers of studies
Weighted index of conditions (mortality)	44 (61%)	[27–29, 32–36, 38, 41, 42, 45, 47, 51–55, 58, 59, 62–64, 66, 69, 70, 72, 73, 75, 77, 79, 81, 84, 86, 87, 89–92, 94, 96–98, 100, 101]
Weighted index of conditions (physical function)	10 (14%)	[62, 63, 65, 66, 69, 84, 92, 94, 95, 97, 98]
Weighted index of conditions (healthcare utilisation or cost)	6 (8%)	[36, 63, 78, 79, 86–88]
Weighted index of conditions (physical function and mortality)	3 (4%)	[38, 80, 97, 98]
Weighted index of body systems	9 (13%)	[34, 41, 49, 50, 53, 57, 69, 76, 82, 83]
Weighted index of medications	7 (10%)	[34, 38, 47, 53, 72, 73, 97, 98]

### Approaches to defining multimorbidity

Less than half of studies (*n* = 31/72, 43%) provided an explicit ‘top level’ definition of multimorbidity (see Table 2). Where a definition was provided most studies (*n* = 27/72, 38%) used an overarching definition of two or more co-existing conditions. Three studies (*n* = 3/72, 4%) that provided a definition used the term comorbidity to describe what is conventionally now described as multimorbidity (i.e. coexistence of two or more long-term conditions without a specific focus on an index condition) [66, 75, 80]. Furthermore, heterogeneity existed in how the two or more conditions were defined, with stipulations provided on the type of conditions of interest based on the length of time living with a condition [37, 48, 52, 60, 70, 71, 78, 79, 88, 94–96], propensity for acute decompensation [37] or impact on care requirements [43, 46, 52, 66, 81] taken into account by 16 (*n* = 16/72, 22%) studies. Buurman and colleagues made the differentiation between acute and chronic multimorbidity, whereby an individual with at least one acute condition alongside a long-term condition was defined as acute multimorbidity, and two or more long-term conditions as chronic multimorbidity [37].

A small number of studies identified multimorbidity through the presence of particularly impactful combinations of conditions. Qualifying comorbidity sets (QCS) were used in three studies to identify multimorbidity within hospital patients admitted with surgical conditions [78, 79, 88]. QCS comprise specific groupings of conditions to identify multimorbidity, identified as being specifically high-risk combinations for predicting mortality. Whilst only a small number of studies have utilised these approaches, the basis for these approaches provides insight for how others could similarly characterise multimorbidity as specific combinations of conditions.

### Approaches to characterising multimorbidity

Weighted indices were the most common approach to characterising multimorbidity (*n* = 54/72, 75%), with simple counts used in fewer studies (*n* = 27/72, 38%). Of the studies that utilised weighted indices, weighted indices of conditions (*n* = 46/72, 64%) were the most common index used to characterise multimorbidity, followed by weighted indices of body systems (*n* = 9/72, 13%) (see Supplementary information 6, Table 2). There were multiple approaches to applying weights which added another source of between-study heterogeneity. Of those studies applying weighting, the most common approach was to apply weights based on the scale of association of a condition with mortality (*n* = 44/72, 61%) (see Table 3).

The Charlson Comorbidity Index (CCI) was the most commonly used weighted index (*n* = 40/72, 56%), but there was heterogeneity in how the CCI was operationalised (see Supplementary Information 6, Table 3). A total of six different versions of the CCI were used which included variation in the number of conditions used (ranging from 12 to 23), with 19 individual conditions the most common approach. The CCI score was used to determine presence or severity of multimorbidity in 19 studies (*n* = 19/72, 26%). Thresholds varied across studies, with 12 different thresholds used to denote either presence or severity of multimorbidity.

The list of conditions used to define multimorbidity was provided in 57 studies (*n* = 57/72, 79%) (see Table 2). A further four studies (*n* = 4/72, 6%) that used multiple approaches to characterise multimorbidity in the same population, provided a list of conditions for some, but not all, approaches. The number of conditions considered when determining the presence of multimorbidity was highly heterogeneous ranging from 2 to 285 (see Table 2).

### Aims of studies and outcomes considered

Most studies aimed to explore the association of multimorbidity with an outcome (*n* = 59/72, 82%). The next most common aim was to assess the validity of the measure used to characterise multimorbidity (*n* = 16/72, 22%) (see Table 2). Two general approaches existed to assessing validity, either using the same approach on a subset of the study population or comparing the approach to other published approaches for characterising multimorbidity within the same population.

Outcomes studied can be classified into those related to: baseline health; the index healthcare event (e.g. hospital admission); long-term outcomes following index healthcare event. Mortality was the most frequently studied outcome, with inpatient mortality an outcome of interest in 13 studies (*n* = 13/72, 18%) and mortality after index healthcare event an outcome of interest in 24 studies (*n* = 24/72, 33%) (see Table 4). There was heterogeneity between studies in how outcomes were measured, for instance variation in the length of follow-up for mortality data from 2 days in-hospital [74] to 5 years post-discharge [69]. Outcomes related to the wider impact of multimorbidity on an individual’s health or healthcare services were studied in fewer populations. Association of multimorbidity with baseline physical function was studied in seven studies (*n* = 7/72, 10%), health-related quality of life in three studies (*n* = 3/72, 4%), and healthcare expenditure in three studies (*n* = 3/72, 4%).

**Table 4:** Outcomes considered by included studies within the scoping review

Studied outcome of interest in association with multimorbidity	Number of study populations	Reference numbers of studies
*Baseline health status*		
Physical function	7	[27, 35, 36, 40, 63, 77, 80]
Presence of geriatric syndromes	3	[38, 54, 101]
Health-related quality of life	3	[36, 91, 93]
Nutritional status	2	[27, 77]
Mental health	2	[36, 44]
*Index healthcare event*		
Inpatient mortality	13	[35, 45, 49, 51, 59, 70, 71, 74, 75, 79, 92, 96, 98]
Length of stay	9	[29, 32, 35, 51, 64, 78, 79, 90, 98]
ITU admission	4	[65, 71, 92, 96]
Hospital admission	1	[55]
Healthcare expenditure	1	[91]
*Long-term outcomes after index healthcare event*		
Mortality	24	[28, 32, 34, 41, 42, 45, 49, 53, 55, 58, 65, 67, 69, 70, 73, 74, 78, 84, 85, 92, 94, 95, 97, 100]
Discharge destination	5	[57, 78, 79, 97, 98]
Measure of functional recovery	4	[61, 62, 76, 89]
Emergency Department reattendance	2	[33, 67]
Healthcare expenditure	2	[43, 78]
Requirement of medical equipment on discharge	1	[78]
General health status	1	[81]

### Account of complexity

Only 12 studies (*n* = 12/72, 17%) explicitly mentioned complexity with a further 22 (*n* = 22/72, 31%) implicitly referring to aspects of complexity (see Table 5). Of the 12 studies that explicitly referred to complexity, this was only a major part of the analysis and discussion in seven (*n* = 7/72, 10%) [32, 35, 44, 46, 81–83]. A number of different approaches to modelling complexity were observed, with no single unifying approach, providing another source of between-study heterogeneity. Three studies defined complexity through the inclusion of specific conditions, such as the use of geriatric syndromes and subjective symptoms by Clerencia-Sierra and colleagues [44, 82, 83]; two studies described complexity in health and care of individuals with multimorbidity [32, 81]; one study described complexity in relation to outcomes [46]; one study described complexity through juxtaposing frailty and multimorbidity, and the importance of function when considering complexity [35].

**Table 5:** Approaches to characterising complexity

Approach to characterising complexity	Total number of study populations	Reference numbers of studies
Definition incorporates complexity through selection of conditions of interest	18	[25, 32, 34, 40, 44, 46, 48, 58, 59, 61, 62, 65, 67, 76, 82, 83, 90, 91, 93, 94]
Definition incorporates wider elements of health in its operationalisation e.g. biochemical markers	5	[50, 62, 63, 80, 93, 94]
Identifies a population of interest termed “complex” or “severe” multimorbidity	6	[60, 68, 79, 81–83]
Study interested in complex interactions between conditions	5	[33, 35, 44, 57, 78]
Study interested in the impact on an individual’s capacity to manage health or health-reported quality of health	6	[39, 42, 54, 77, 87, 89]

When taking a wider view of complexity, including those studies which implicitly accounted for complexity, the majority (*n* = 18/34, 53%) accounted for complexity through inclusion of specific conditions (see Table 5). Conditions were often selected due to their impact on function and disability or association with increased healthcare utilisation [35, 40, 48, 58, 59, 67, 82, 83, 87, 90, 91]. For example, in the study by Chen et al. [43] on multimorbidity and healthcare expenditure, 33 conditions were chosen due to their impact on the burden of disease and disability in older adults. These conditions included dementia, hearing loss and Parkinson’s disease, with impact determined by calculating the disability-adjusted life years associated with each condition.

## Discussion

We undertook a scoping review of published quantitative studies to review approaches to characterising multimorbidity in older people accessing hospital care. We explored the extent to which complexity of conditions and care were accounted for in these studies, and identified the outcomes assessed in relation to multimorbidity. Our review identified 75 papers reporting on 72 unique study populations, and highlights that within the hospital setting multimorbidity has not been robustly or consistently characterised. Less than half of studies provided the reference definition used to characterise multimorbidity, limiting how effectively conclusions drawn from any single study can be understood in a wider context. Weighted indices were the most frequently utilised approach to operationalise multimorbidity, with the Charlson Comorbidity Index the most commonly used index. There was considerable heterogeneity between studies in how the CCI was operationalised, again limiting comparability. Complexity was rarely addressed, with only 12 studies explicitly exploring this.

### Characterising multimorbidity and appreciating complexity

The field of multimorbidity research is in its relative infancy and has until recently faced challenges in determining how to define and operationalise multimorbidity. The top-level definition of two or more long-term conditions has now been widely adopted and this is encouraging [1]. However, how this is operationalised in different research studies is not always clear and depends on the setting and purpose of the study [1]. Arguably most useful is the promotion of a need to explicitly define multimorbidity within published work, so the field can interpret findings in a wider context [4]. We report that less than half of studies of multimorbidity in older people accessing hospital care provided a definition of multimorbidity within their work. However, encouragingly, an increase in the reporting of a definition is noted with evidence in our review that recently published studies were more likely to report a definition and approach to operationalisation than studies described in the Ho et al. review. Studies included within the Ho et al. review account for 53% (*n* = 38/72) of the included study populations in our review, and in these only 32% (*n* = 12/38) defined multimorbidity. In studies published subsequently, 56% (*n* = 19/34) reported a definition. This increase is promising, but there remains a considerable number of studies where a reference definition of multimorbidity is not reported.

Alongside a clearly stated top-level definition, understanding the list of chronic conditions from which studies define multimorbidity is important. Within this review, the number of conditions that studies used ranged from 2 to 285, with approximately a third of studies utilising approaches that contained 16–30 conditions. This between-study heterogeneity impacts on the ability to make comparisons across studies, which remains a critical challenge for the field. Work by MacRae and colleagues has shown large differences in the prevalence of multimorbidity estimated when different lists of conditions are used to operationalise multimorbidity in the same population [102]. A Delphi consensus study has been undertaken to develop international consensus on the definition and measurement of multimorbidity [5] and the principles for this have subsequently been adapted for use in the hospital context [6].

The adoption of the top-level definition of two or more long-term conditions was widely observed in studies in our review; however, far fewer considered complexity of conditions, with this less well defined. The limited appreciation of complexity of conditions is arguably of greater significance in older people where the majority will have multimorbidity when the standard top-level definition is applied, and identification of a more complex subset is likely to hold clinical relevance [103]. Approaches to defining complexity vary dependent on the reason it was being considered [104]. An often-quoted approach for defining complexity is by Harrison and colleagues, whereby complex multimorbidity is the presence of three or more long-term conditions from three or more body systems (termed “3+ from 3+”) [103]. They argue that inclusion of body systems increases the likelihood that an individual will have more interactions with multiple different healthcare professionals from a range of specialties compared to those individuals with a similar number of conditions but from the same body system [103, 105]. Despite being one of the most widely utilised approaches in the multimorbidity field as a whole, no studies were identified in our review that had used this approach in a hospital setting [104]. Other studies have modelled approaches using increasing numbers of conditions to identify more “severe” groups, but with limited description of how this equates to complexity [2]. The relationships between different individual conditions is thought to contribute to this complexity, with the combination of physical and mental health conditions known to be particularly detrimental to overall health outcomes [12]. The complexity of these relationships can be hard to appreciate through simple counts of conditions and weighted indices [12]. Although approaches that characterise and account for complexity exist, there is currently a lack of consensus amongst researchers in determining what approach to take when studying complexity as it relates to multimorbidity.

Only seven studies identified in this review explicitly described complexity, with heterogeneity in approaches [36, 39, 48, 50, 85–87]. Complexity and severity are often used interchangeably, with the weighting of conditions towards aspects of complexity the most utilised approach [36, 50, 86, 87]. Understanding how weights are applied is challenging, with what determines ‘severity’ or ‘complexity’ not always clear [36, 86, 87]. Complexity in older people specifically has been described through the inclusion of frailty or geriatric syndromes within the characterisation of multimorbidity and provides an approach by which the impact on function or inclusion of important subjective symptoms (such as pain) can be included [39, 48]. Interaction between medical conditions and complexity of care requirement as described by Yarnall and colleagues was rarely considered in studies identified within this review [20, 85]. The solitary study that explicitly addressed the interaction of conditions and complexity of care was undertaken by Shakib and colleagues [85]. They developed an interventional study based on a multidisciplinary outpatient model of care for older patients with multimorbidity, and referred to complexity in both the care individuals experience and the number and combinations of conditions they have [85].

That few studies describe complexity in older adults living with multimorbidity accessing hospital care highlights a need for further study. Although there are approaches, such as Harrison’s [103], which are more widely recognised in the multimorbidity field, these are yet to be employed to understand complexity within older adults with multimorbidity accessing hospital care. It is unclear whether these approaches will uncover different meaningful patterns of multimorbidity [103], but further work exploring the interaction between complexity and multimorbidity, through recognised and novel approaches, is required to determine if more meaningful groups of individuals can be identified who will benefit from tailored management of their multimorbidity.

### Outcomes for older adults with multimorbidity

Summarising outcomes studied in relation to multimorbidity was an expressed aim of this scoping review. Most studies (*n* = 59/72, 82%) identified explored associations between multimorbidity and different outcomes. Mortality, whether inpatient or following discharge, was the most frequently studied outcome, with many reporting an association between the presence of multimorbidity and increased rates of death: this mirrors the findings of previous reviews across healthcare settings [9].

Whilst mortality and other hospital stay-related metrics such as length of stay were the most frequently studied outcomes, other outcomes potentially of more significance to the individual, such as health-related quality of life, have also been studied [36, 91, 93, 106]. For the individual, treatment burden, the effort required to manage one’s health, is a key metric of the impact of multimorbidity [107, 108]. Differing models exist for measuring treatment burden, including the cumulative complexity model [109] and the Multimorbidity Treatment Burden Questionnaire (MTBQ) [107], but often proxy measures have been used to attempt to quantify treatment burden. No studies within this scoping review explicitly measured treatment burden, as a standalone analysis or in relation to wider features of complexity. Treatment burden is important for the individual and wider healthcare structures, as it is the patient population with treatment burden who are described as those likely to benefit from targeted intervention [20, 110]: this gap needs addressing.

Government bodies are recognising the challenge that multimorbidity presents for healthcare systems [3, 111, 112]. Real-world outcomes are of increased interest, but it is not clear what these are. The European Commission released a call for multimorbidity research in “Horizon Europe: Work Programme 2023–2025, Health”, challenging researchers to explore implementation science approaches for managing multimorbidity [3]. Within their call, they advocated the need to develop “an appropriate strategy for measuring implementation research outcomes and real-world effectiveness outcomes and indicators”. Whilst the acknowledgement of the need to address multimorbidity is welcome, to aid the development of targeted interventions more study is required to ensure that the approaches for characterising multimorbidity account for real-world issues including complexity. This will ensure that multimorbidity interventions reach the populations in most need of tailored approaches to care.

## Strengths and limitations

A strength of this scoping review is its focus on multimorbidity among older adults accessing hospital care; a growing population with complex care needs which are currently not being adequately met [4]. We explicitly sought to describe studies that characterised multimorbidity rather than comorbidity [1]. Many studies initially identified as potentially eligible were subsequently excluded owing to their comorbidity focus. It was a common approach for organ-specific research to describe the CCI in association with a specific problem, such as a subset of hip fractures; but this has limited applicability for the wider multimorbidity field. Through taking this explicit approach we have provided an overview specific to multimorbidity, from which clear gaps can be observed and prioritised.

Alongside its key strengths, the review also has limitations. Firstly, we chose to focus on peer-reviewed literature; whilst this approach provides a degree of quality assurance potentially relevant studies within grey-literature may have been missed. The Ho et al. review did include grey literature, but only one of the 566 papers in their review was from this source and would not meet wider inclusion criteria for our study. This highlights the limited benefit to reviewing grey literature for our specific research question and supports our decision for exclusion. Through focusing on quantitative studies, we acknowledge that relevant learning from qualitative research is not captured. However, our a priori decision to focus on quantitative research was driven by the need to understand the characteristics of multimorbidity at a population level. We also acknowledge that our focus on older adults overlooks younger adults living with multimorbidity, who may also experience complexity. However, patterns and characteristics of multimorbidity are known to vary by age justifying our specific focus.

### Evidence gaps

Our review highlights a number of key evidence gaps in current understanding of multimorbidity in older adults accessing hospital care. Firstly, how multimorbidity is measured and characterised within studies of older adults accessing hospital care is poorly stated and highly inconsistent. The advice which Ho et al. [4] promote in their associated systematic review, to provide consistent reporting of measured definitions is applicable here too, along with the need to provide justification for the selection of specific conditions when operationalising multimorbidity. Reliance on weighted indices to characterise multimorbidity across the studies identified means that broader understanding of prevalence of long-term conditions and how multimorbidity changes within this population by age, sex and socioeconomic position is significantly lacking and further study is needed [4, 113]. At a European level, the impact of multimorbidity on disadvantaged populations was highlighted as a priority area of research, but little research has been undertaken to describe this association within older hospital populations [3]. Novel statistical approaches have been developed to describe patterns of multimorbidity [114], and whilst these are increasingly being employed in primary care and community-based data to describe multimorbidity, we found that they have been seldom employed within the hospital setting.

A high proportion of hospitalised older adults are likely to have complex multimorbidity (irrespective of how this is defined), by virtue of the fact they are a population with high prevalence of multimorbidity who are engaging with hospital-based services. Describing this complexity will be important when developing approaches that aim to better meet the needs of this group and improve their experiences of hospital care and outcomes. Strategies for managing multimorbidity are being explored, such as multimorbidity clinics [115], but how the populations that would gain most from explicit management of their multimorbidity would be identified has not yet been established and is a key research priority. Furthermore, understanding the complex care pathways that older individuals with multimorbidity experience through the hospital setting, and beyond into the community and care sector is also limited; improving this is of importance to the individual and healthcare structures [3, 13]. If complexity and “complex multimorbidity” are being studied, we believe it would be beneficial if they are named and described explicitly, to aid knowledge sharing across the field.

In this scoping review, the majority of studies were from just five countries (*n* = 42/72, 58%). Although 36 studies (*n* = 36/72, 48%) were conducted in European countries, only 15 countries in Europe were represented. As demographic characteristics and healthcare structures differ across the continent, and projections of multimorbidity prevalence vary too, there is arguably a need to better understand variations in the experiences of older adults with multimorbidity accessing hospital care across countries and healthcare systems [116]. Improving knowledge across all of Europe increases possibilities for shared learning to occur to hopefully improve care and outcomes for all older adults with multimorbidity.

## Conclusion

Multimorbidity is increasingly recognised as an important and growing challenge for healthcare, but our understanding of multimorbidity in older adults accessing hospital care is currently limited. Our review highlights heterogeneity in how multimorbidity has been characterised in older adults accessing hospital care, with variations in definition and conditions of interest, and limited consideration of complexity. Further research is required to understand patterns of multimorbidity in this group adequately accounting for complexity in both combinations of conditions and care needs in order to provide appropriately tailored care, and to ensure interventions developed as part of wider government multimorbidity strategies meet the needs of this growing population.

## Supplementary Information

Below is the link to the electronic supplementary material.

Supplementary file1 (DOCX 86 KB)

Supplementary file2 (DOCX 45 KB)

Supplementary file3 (DOCX 21 KB)

Supplementary file4 (XLSX 12 KB)

Supplementary file5 (DOCX 169 KB)

Supplementary file6 (DOCX 263 KB)

## Data Availability

Not applicable.
