# Supplementary material for: Approaches to characterising multimorbidity in older people accessing hospital care: a scoping review
Source: Eur Geriatr Med. 2025 Mar 1;16(4):1099–113. doi: 10.1007/s41999-025-01166-3 (PMC12378491; doi:10.1007/s41999-025-01166-3)
Supplement: Supplementary file 2 — Supplementary file2 (DOCX 45 KB) [file 41999_2025_1166_MOESM2_ESM.docx]

**Approaches to characterising multimorbidity in older people accessing hospital care**: **a scoping review**

Jonathan G Bunn^1,2^, Lewis Steell^1,2^, Susan J Hillman^1,2^, Miles D Witham^1,2^, Avan A Sayer^1,2^ and Rachel Cooper^1,2^ on behalf of the ADMISSION research collaborative

1. AGE Research Group, Translational and Clinical Research Institute, Faculty of Medical Sciences, Newcastle University, Newcastle upon Tyne, UK

2. NIHR Newcastle Biomedical Research Centre, Newcastle upon Tyne Hospitals NHS Foundation Trust, Cumbria Northumberland Tyne and Wear NHS Foundation Trust and Faculty of Medical Sciences, Newcastle University, Newcastle upon Tyne, UK

**Corresponding Author:**

Rachel Cooper, email rachel.cooper@newcastle.ac.uk

ORCID ID: 0000-0003-3370-5720

**Journal of Submission**: European Geriatric Medicine

**Supplementary Information 2: Scoping Review Protocol**

**Title:** Approaches to characterising multimorbidity in older people accessing hospital care: a scoping review

**Review team:** Jonathan Bunn, Susan Hillman, Rachel Cooper, Lewis Steell

**Aim:**

The aims of this scoping review are threefold, to:

1. Identify approaches that have been taken to characterise multimorbidity in older people accessing hospital care, and the extent to which these approaches take account of the complexity of conditions and care.
2. Summarise the outcomes that have been studied in relation to older people with multimorbidity who access hospital care.
3. Describe key gaps in the literature that need to be addressed to further our understanding of multimorbidity in older adults accessing hospital care.

**Approach**

This review has been developed using the framework for scoping reviews initially proposed by Arksey and O’Malley in 2005 [1], and revised by Levac and colleagues in 2010 [2]. These are detailed below, and each will be discussed in turn through this protocol:

1. Identifying the research question
2. Identifying relevant studies
3. Study selection
4. Charting the data
5. Collating, summarising, and reporting the results

**Identifying the research question**

1. Research question framework

This scoping review will use the CoCoPop framework (Condition, Context and Population) to frame the research question [3]. CoCoPop was utilised in the systematic review by Ho et al which sought to examine variation in the measurement of multimorbidity in community, primary care, care home or hospital settings [4]. My review will seek a similar understanding of the operationalisation of multimorbidity but will look further at how multimorbidity has been operationalised within those aged 65 and over in the hospital setting.

**Table 1: CoCoPop Framework**

| Co-Condition | Multimorbidity (including chronic physical, mental health and infectious conditions). |
| --- | --- |
| Co-Context | Hospital-based setting (inpatient, outpatient and emergency department care) |
| Pop-Population | People aged 65 and over. |

1. Scoping Review Question
   1. What approaches have been taken to characterise multimorbidity in older adults accessing hospital care and to what extent to have these approaches take account of the complexity of multimorbidity at older ages?
   2. What outcomes for older adults with multimorbidity in hospital have been studied?
   3. What key gaps are in the literature that we may be able to address to further our understanding of multimorbidity in older adults?

Multimorbidity is now widely accepted as being two or more conditions, comprising of either chronic physical, mental health or infectious conditions [5]. However, within older adults attempts to identify complexity have been made, which define multimorbidity in differing ways using higher numbers of conditions or involvement of different body systems [6]. This review will draw together and summarise all definitions to gain a wider understanding of attempts to characterise multimorbidity within this population.

For this review hospital care will be defined as inpatient and outpatient secondary or tertiary care, where care is delivered by specialists who are not the individuals usual medical practitioner. Via this definition community hospitals where care can be led or co-provided by primary care will not be included, neither will virtual wards or other forms of hospital at home where the person is managed within their own environment.

**Identifying relevant studies**

1. Search strategy: database and search string

It is recommended that the search strategy for scoping reviews is wide, with scoping reviews commonly identifying relevant studies in both published and grey literature. However, this review will focus on peer-reviewed, published studies, as we expect the majority of papers of relevance will end up published. Quantitative rather than qualitative studies will be reviewed, given opportunities they afford to understand how multimorbidity is characterised at a population level: we recognise there are outcomes that may be better explored using qualitative work.

The systematic review by Ho et al provides reference for this scoping review [4]. Through use of the 566 studies identified in their review, our eligibility criteria will be applied to determine the subset that meet the inclusion criteria for this scoping review. Searches will then be replicated and extended to identify relevant literature after the end date of their initial search, 21/1/2020, to present. The following databases will be used: CINAHL; Ovid (MEDLINE, Embase and PsycINFO); Scopus; Web of science; The Cochrane Library; CAB Direct (Global Health). Searches of ProQuest Dissertations and Theses Global will not be undertaken given the focus of this new review on peer-reviewed literature. To balance sensitivity and specificity of the scoping review of the search, we have decided to limit searches to title, abstract, and keyword fields.

**Table 2: Sample search sting based upon Ho’s searches within MEDLINE**

| **Concept** | **Search string** |
| --- | --- |
| MLTC | 1. (multimorbidit$ or multi-morbidit$ or comorbidit$ or co-morbidit$ or polymorbidit$ or polymorbidit$ or multicondition$ or multicondition$ or "multiple chronic condition$" or "morbidity burden").m_titl. 2. ((multiple or coexisting or co-existing or concurrent or con-current or comorbid or co-morbid) adj2 (disease* or illness* or condition* or diagnos* or morbid*)).m.titl |
| Measurement of MLTC | 1. (measure$ or index or indices or instrument$ or scale$ or "disease count$").mp. [mp=ti, bt, ab, ot, nm, hw, fx, kf, ox, px, rx, ui, sy, ux, mx, tn, dm, mf, dv, dq, tc, id, tm] |
| Combined search | 1. 1 or 2 2. 3 and 4 |
| Limiters | 1. limit 5 to (human and yr="2020 -Current") |

Ho’s search string does have limitations. In particular, the lack of subject search, search of titles only for the multimorbidity concept and the variation of the search between databases. Measurement of multimorbidity is searched using ‘mp’ in medline, but in CINAHL this search occurs only in abstracts. For consistency we will utilise the same searches as Ho but recognise that sensitivity of the search may have been improved by addressing the issues described.

1. *Documenting searches*

Records will be made of all literature searching activities, guided by the PRISMA-S checklist [7], to ensure reproducibility of the search strategy. The PRISMA-S checklist will be made available as a supplementary document.

**Study selection**

Discussions between JB and his wider supervisory group (RC, AAS, SH)) were held around inclusion and exclusion criteria in the early stages of the scoping review process, as per recommended guidelines [2]. Two of the key concepts of the scoping search strategy are what “hospital setting” is, which was described in more detail in the “identifying the research question” section, and how to determine if a study meets the age of interest criteria. From an age perspective, if the study explicitly looks at those 65 years and over it will be included, and if the reported average of the population within a study were identified as 65 years and over it will be included, or if it can be confirmed by other means that the average must lie above 65 years.

**Table 3: Eligibility Criteria**

| **Inclusion Criteria** | **Exclusion Criteria** |
| --- | --- |
| Study type: quantitative studies | Qualitative studies, mixed methods studies, study protocols, literature reviews, editorials, and commentaries. |
| Age of interest: studies included that look explicitly at people aged ≥65 years or where the reported average age of the study population is ≥65 years. | Studies that have no age analysis to determine the age distribution of the population or where the reported average of the study population is <65 years. |
| Hospital setting: emergency department, inpatient and outpatient secondary or tertiary care, where care is delivered by specialists who are not the individuals’ usual medical practitioner. | Studies that focus on comorbidity; whereby there is an index condition of focus within the study. |
| Peer-reviewed papers | Community based studies, cohort studies, community hospitals setting, virtual wards or other forms of hospital at home |
| English language | Studies that have not been peer reviewed, grey literature |
| Study Date:   - Studies included in the Ho et al searches were from inception of the database to the 21/1/2020. - Replication and extension of Ho et al searches from 21/1/2020 to 7/9/23. |  |

Collating, summarising, and reporting the results

Extracted data from studies will be charted in tables to allow for comparison and analysis of findings and themes across studies. The charted data will include information, at minimum limited to:

- Study ID, title, and journal of publication.
- Author
- Year of publication
- Country of study
- Sample characteristics: demographic data (male/female split, size of population)
- Age-measure of average within the study, or age frequency information
- Type of hospital care being accessed
- Data source
- Definition of MLTC used
- Number of conditions of interest
- Study aim
- Method of characterising MLTC (count-based approach, indices)
- Account of complexity
- Outcomes of interest
- Key findings e.g. prevalence of MLTC, MLTC clusters

As the main role of this review is to determine the ways in which MLTC has been operationalised in older hospitalised people particular focus will be paid around the age structure of the study population, and how exactly MLTC has been defined and operationalised for the purposes of the study, rather than the reported findings.

Data extraction and quality assessment

The findings from the scoping review will be summarised according to identified key themes. The review will be structured to identify the current knowledge base, focussing on the methods by which MLTC has been defined within the population of interest, and identifying gaps for future research.

Reporting

PRISMA-ScR is a checklist that has been developed for scoping reviews and will be completed as a supplementary document to ensure the review process is transparent and comprehensive and adheres to recommended guidelines [7].

References

1. Arksey H and O'Malley L. Scoping studies: towards a methodological framework. International Journal of Social Research Methodology. 2005. **8**(1): p. 19-32 DOI: <https://doi.org/10.1080/1364557032000119616>.

2. Levac D, Colquhoun H, and O'Brien KK. Scoping studies: advancing the methodology. Implementation Science. 2010. **5**(1): p. 69 DOI: <https://doi.org/10.1186/1748-5908-5-69>.

3. Munn Z, et al. Methodological guidance for systematic reviews of observational epidemiological studies reporting prevalence and cumulative incidence data. Int J Evid Based Healthc. 2015. **13**(3): p. 147-53 DOI: <https://doi.org/10.1097/xeb.0000000000000054>.

4. Ho ISS, et al. Examining variation in the measurement of multimorbidity in research: a systematic review of 566 studies. The Lancet Public Health. 2021. **6**(8): p. e587-e597 DOI: <https://doi.org/10.1016/S2468-2667(21)00107-9>.

5. The Academy of Medical Sciences. Multimorbidity: A Priority For Global Health Research. 2018.

6. Harrison, C., et al. Examining different measures of multimorbidity, using a large prospective cross-sectional study in Australian general practice. BMJ Open. 2014. **4**(7): p. e004694 DOI: 10.1136/bmjopen-2013-004694.

7. Tricco AC, et al. PRISMA Extension for Scoping Reviews (PRISMA-ScR): Checklist and Explanation. Ann Intern Med. 2018. **169**(7): p. 467-473 DOI: <https://doi.org/10.7326/m18-0850>.
