# Supplementary material for: Approaches to characterising multimorbidity in older people accessing hospital care: a scoping review
Source: Eur Geriatr Med. 2025 Mar 1;16(4):1099–113. doi: 10.1007/s41999-025-01166-3 (PMC12378491; doi:10.1007/s41999-025-01166-3)
Supplement: Supplementary file 3 — Supplementary file3 (DOCX 21 KB) [file 41999_2025_1166_MOESM3_ESM.docx]

**Approaches to characterising multimorbidity in older people accessing hospital care**: **a scoping review**

Jonathan G Bunn^1,2^, Lewis Steell^1,2^, Susan J Hillman^1,2^, Miles D Witham^1,2^, Avan A Sayer^1,2^ and Rachel Cooper^1,2^ on behalf of the ADMISSION research collaborative

1. AGE Research Group, Translational and Clinical Research Institute, Faculty of Medical Sciences, Newcastle University, Newcastle upon Tyne, UK

2. NIHR Newcastle Biomedical Research Centre, Newcastle upon Tyne Hospitals NHS Foundation Trust, Cumbria Northumberland Tyne and Wear NHS Foundation Trust and Faculty of Medical Sciences, Newcastle University, Newcastle upon Tyne, UK

**Corresponding Author:**

Rachel Cooper, email rachel.cooper@newcastle.ac.uk

ORCID ID: 0000-0003-3370-5720

**Journal of Submission**: European Geriatric Medicine

**Supplementary Information 3: Summary of electronic database search strings**

| Search | Search string | Number of hits |
| --- | --- | --- |
| Web of science | ((((TI=(measure* or index or indices or instrument* or scale*))) AND TI=(multimorbidit* or multi-morbidit* or comorbidit* or co-morbidit* or polymorbidit* or poly-morbidit* or multicondition* or multicondition* or 'multiple chronic condition*' or 'morbidity burden' )) OR TI=((multiple or coexisting or co-existing or concurrent or con-current or comorbid or co-morbid)NEAR/2 (disease* or illness* or condition* or diagnos* or morbid*)))  Limit 2020-current  Excluded meeting abstracts | 3481 |
| Scopus | TITLE(multimorbidit*OR multi-morbidit *OR comorbidit* OR co-morbidit* OR polymorbidit* OR poly-morbidit* OR multicondition* OR multicondition* OR "multiple chronic condition*" OR "morbidity burden" OR ((multiple OR coexisting OR co-existing OR concurrent OR con-current OR morbid OR co-morbid) W/2 (disease* OR illness* OR condition* OR diagnos?s OR morbid*))) AND TITLE (measure* OR index OR indices OR instrument* OR scale* OR "disease counts") AND PUBYEAR> 2019 AND PUBYEAR <2024 AND (LIMIT-TO (EXACTKEYWORD, "Human")) AND (LIMIT-TO (LANGUAGE, "English")) | 441 |
| Ovid (Medline, PsychInfo, EMBASE) | 1. (multimorbidit$ or multi-morbidit$ or comorbidit$ or co-morbidit$ or polymorbidit$ or polymorbidit$ or multicondition$ or multicondition$ or "multiple chronic condition$" or "morbidity burden").m_titl. 2. ((multiple or coexisting or co-existing or concurrent or con-current or comorbid or co-morbid) adj2 (disease* or illness* or condition* or diagnos* or morbid*)).m titl 3. (measure$ or index or indices or instrument$ or scale$ or "disease count$").mp. [mp=ti, bt, ab, ot, nm, hw, fx, kf, ox, px, rx, ui, sy, ux, mx, tn, dm, mf, dv, dq, tc, id, tm] 4. 1 or 2 5. 3 and 4   Limited to full text, humans, english language, 2020 to present. | 1206 |
| CINAHL | 1. MM (multimorbidit* or multi-morbidit* or comorbidit* or co-morbidit* or polymorbidit* or poly-morbidit* or multicondition* or multicondition* or “multiple chronic condition*” or “morbidity burden” or ((multiple or coexisting or co-existing or concurrent or con-current or comorbid or co-morbid) N2 (disease* or illness* or condition* or diagnos* or morbid*)))  2. AB (measure* or index or indices or instrument* or scale*)  3. 1 AND 2  Linked full text, human, English language | 15 |
| Cochrane Library | ((measure or index or indices or instrument or scale or “disease count*”)):ti AND (multimorbidity or multi-morbidity or comorbidity or co-morbidity or polymorbidity or poly-morbidity or multicondition or multicondition or 'multiple chronic conditions' or 'morbidity burden' or ((multiple or coexisting or co-existing or concurrent or con-current or comorbid or co-morbid) NEAR/2 (disease or illness or condition or diagnosis or morbid))):ti  Limited to 2020 to present | 24 |
| CAB Direct  Global Health | Title:((measure* or index or indices or instrument* or scale*)) AND title:(multimorbidity* or multi-morbidit* or comorbid* or co-morbidit* or polymorbidit* or poly-morbidit* or multicondition* or multicondition* or ‘multiple chronic condition*’ or ‘morbidity burden’) OR title:(((multiple or coexisting or co-existing or concurrent or con-current or comorbid or co-morbid)NEAR/2 (disease* or illness* or condition* or diagnos* or morbid*))) AND ex:[2020-01-01 TO *]  Refinements:  Language=English  AND Organism Descriptors=man | 22 |
