# Supplementary material for: Approaches to characterising multimorbidity in older people accessing hospital care: a scoping review
Source: Eur Geriatr Med. 2025 Mar 1;16(4):1099–113. doi: 10.1007/s41999-025-01166-3 (PMC12378491; doi:10.1007/s41999-025-01166-3)
Supplement: Supplementary file 5 — Supplementary file5 (DOCX 169 KB) [file 41999_2025_1166_MOESM5_ESM.docx]

**Approaches to characterising multimorbidity in older people accessing hospital care**: **a scoping review**

Jonathan G Bunn^1,2^, Lewis Steell^1,2^, Susan J Hillman^1,2^, Miles D Witham^1,2^, Avan A Sayer^1,2^ and Rachel Cooper^1,2^ on behalf of the ADMISSION research collaborative

1. AGE Research Group, Translational and Clinical Research Institute, Faculty of Medical Sciences, Newcastle University, Newcastle upon Tyne, UK

2. NIHR Newcastle Biomedical Research Centre, Newcastle upon Tyne Hospitals NHS Foundation Trust, Cumbria Northumberland Tyne and Wear NHS Foundation Trust and Faculty of Medical Sciences, Newcastle University, Newcastle upon Tyne, UK

**Corresponding Author:**

Rachel Cooper, email rachel.cooper@newcastle.ac.uk

ORCID ID: 0000-0003-3370-5720

**Journal of Submission**: European Geriatric Medicine

**Supplementary Information 5: A table of key features of included papers ordered alphabetically by first author’s surname and year of publication**

| Authors and year of publication | Study Population   - Sample size (no. of adults) - Age (mean ± SD to nearest whole no.) - Sex (% female, to nearest whole no.) | Study Setting   - Hospital setting [emergency department (ED) attendance inpatient/ outpatient /not clear] - Country | Study Aim | Data Source | Definition of multi-morbidity provided | Type of multi-morbidity measure | Justification of conditions included in definition of multi-morbidity | Number of conditions of interest | Account of complexity |
| --- | --- | --- | --- | --- | --- | --- | --- | --- | --- |
| Amasene, Medrano et al. 2022 [1] | 597 adults  84±7 years  50% female | Medical inpatients, University Hospital  [Inpatient]  Spain | Association of multimorbidity with an outcome | Medical records and administrative databases | No | Weighted index of conditions (CCI-A) | Yes | 17 | No |
| Afken, Lichtenberg et al. 1998 [2] | 667 adults  77±8 years  65% female | Rehabilitation inpatients,  Rehabilitation Hospital [Inpatient]  USA | Association of multimorbidity with an outcome | Medical records and administrative databases | No | Weighted index of conditions (DCCI) | Yes | 17 | No |
| Aubert, Fankhauser et al. 2019 [3] | 33,871 adults  Median 68 (IQR 56-78) years  43% female | Medical inpatients,  University Hospital [Inpatient]  Switzerland | Association of multimorbidity with an outcome | Medical records and administrative databases | Yes | Count of conditions  Weighted index of conditions (DCCI, EVWCI) | Yes-for count  No-for indices | Count: 285  Deyo-CCI: not reported  EVWCI: not reported | Yes-implicit |
| Bahat, Tufan et al. 2013 [4] | 274 adults  74.±7 years  0% female | Outpatients, Geriatric Medicine Department [Outpatient]  Turkey | Prevalence or burden of multimorbidity | Self-report | No | Count of conditions | No | Not reported | No |
| Bahat, Tufan et al. 2014 [5] | 515 adults  73±7 years  100% female | Outpatients, Geriatric Medicine Department  [Outpatient]  Turkey | Prevalence or burden of multimorbidity | Self-report | No | Count of conditions | No | Not reported | No |
| Bahrmann, Benner et al. 2019 [6] | 307 adults  80±6 years  51% female | Non-trauma patients, Tertiary Hospital [Inpatient]  Germany | Performance of the measure, to itself or others, in characterising multimorbidity | Self-report  Medical records and administrative databases | No | Weighted index of conditions (CCI) | Yes | 19 | No |
| Bayliss, Ellis et al. 2015 [7] | 12,200 adults aged ≥65 years with multimorbidity identified. Two outcomes considered in analyses: inpatient admission or ED attendance. For both outcomes all individuals categorised to receive primary care (PC) only, PC or specialty care (SC) or SC only.  In all categories mean age (SD) ~76 (6), 54-56% female. | Inpatient admissions and ED attendances  [ED/Inpatient]  USA | Study of populations with multimorbidity | Medical records and administrative databases | No | Weighted index of conditions  (Quan adjusted-ECI) | Yes | 30 | No |
| Beloosesky, Weiss et al. 2011[8] | 212 adults  81±7 years  62% female | Geriatric inpatients, University Hospital  [Inpatient]  Israel | Performance of the measure, to itself or others, in characterising multimorbidity | Self-report  Medical records and administrative databases | No | Weighted index of medications (MDBI)  Weighted index of conditions (CCI)  Weighted index of body systems (CIRS-G) | Yes | MDBI=20  CCI=19  CIRS-G=14 organ systems | No |
| Bernard, Inderjeeth et al. 2016 [9] | 306 adults  82±8 years  58% female | Rehabilitation patients, secondary care hospital  [Inpatient]  Australia | Association of multimorbidity with an outcome | Medical records and administrative databases | No | Weighted index of conditions (CCI) | Yes | CCI=19 | No |
| Briongos-Figuero, Cobos-Siles et al. 2020 [10] | 305 adults  88±5 years  59% female | Medical admissions, University hospital  [Inpatient]  Spain | Association of multimorbidity with an outcome | Self-report  Medical records and administrative databases | No | Weighted index of conditions (CCI, CRG) | Yes | CCI=19  CRG= not reported | Yes-explicit |
| Buurman, Frenkel et al. 2016 [11] | 639 adults,  78±8 years  54% female | Medical admissions, 3 teaching hospitals  [Inpatient]  Netherlands | Prevalence or burden of multimorbidity | Medical records and administrative databases. | Yes | Count of conditions | Yes | 23 (16 chronic conditions, 7 acute conditions) | Yes-implicit |
| Canaslan, Ates Bulut et al. 2022 [12] | 366 adults  76±7 years  68% female | Geriatric inpatients, University Hospital  [Inpatient]  Turkey | Association of multimorbidity with an outcome | Not reported | No | Weighted index of conditions (CCI, ECI, GIC)  Weighted index of medications (MCI) | Yes | CCI=19  ECM=30  GIC=15  MCI=20 chronic diseases | Yes-implicit |
| Canevelli, Raganato et al. 2020 [13] | 185 adults  75±8 years  60% female | Memory clinic, University of Rome,  [Outpatient]  Italy | Study of populations with multimorbidity | Self-report  Medical records and administrative databases. | Yes | Count of conditions | No | 18 | Yes-explicit |
| Capisizu, Aurelian et al. 2015 [14] | 80 adults  72±10 years  80% female | Geriatric inpatients, Hospital  [Inpatient]  Romania | Association of multimorbidity with an outcome | Self-report  Medical records and administrative databases. | Not reported | Count of conditions | No | 16 | No |
| Chan, Maddison et al. 2021 [15] | 693 adults  82±8 years  57% female | Geriatric rehabilitation wards, Tertiary Hospital  [Inpatient]  Australia | Association of multimorbidity with an outcome | Not reported | Yes | Weighted index of conditions (CCI, CCI-A)  Weighted index of body systems (CIRS-G, CIRS-G severity index) | Yes | CCI=19  CCI-A=19  CIRS-G=14  CIRS-G severity index=14 | No |
| Chan, Luk et al. 2014 [16] | 2050 adults  81±7 years  60% female | Geriatric Day Hospital [Outpatient]  China | Performance of the measure, to itself or others, in characterising multimorbidity | Medical records and administrative databases. | No | Weighted index of conditions (CCI) | Yes | CCI=14 | No |
| Chen, Chen et al. 2018 [17] | 28,613 adults  Median age between 65 and 69 years (no reported exact mean or median)  51% female | Inpatient or outpatient attendances [Inpatient/  Outpatient]  China | Association of multimorbidity with an outcome | Medical records and administrative databases. | Yes | Count of conditions | Yes | 33 | Yes-implicit |
| Cheng, Inder et al. 2021 [18] | 185 adults  70±7 years  45% female | Tertiary Hospital,  [Unknown]  China | Study of populations with multimorbidity  Association of multimorbidity with an outcome | Self-report | Yes | Count of conditions | No | Not reported | Yes-implicit |
| Chou, Huang et al. 2020 [19] | 2481 adults  72±16 years  47% female | Inpatients with infection,  Tertiary Hospital  [Inpatient]  Taiwan | Association of multimorbidity with an outcome | Medical records and administrative databases | No | Weighted index of conditions (CCI) | Yes | 16 | No |
| Chow and Wong 2014 [20] | 281 adults  Median: 76 years (range 60-92)  52% female | Multimorbidity patients (with one of respiratory, cardiac, type 2 DM and renal disease) discharged from general hospital  [Inpatient]  China | Study of populations with multimorbidity | Medical records and administrative databases | Yes | Count of conditions | Yes | 4 | No |
| Chua, Pan et al. 2023 [21] | 2750 adults  Median 78 years (IQR 72 to 84)  57% female | Trauma patients, Trauma centres  [ED]  Singapore | Association of multimorbidity with an outcome | Medical records and administrative databases | No | Weighted index of conditions (CCI)  Weighted index of conditions and medications (CPS) | Yes (CCI)  No (CPS) | CCI=16  CPI=Not reported | No |
| Clerencia-Sierra, Calderón- Larrañaga et al. 2015 [22] | 924 adults  82±7 years  57% female | Geriatric inpatients, University Hospital  [Inpatient]  Spain | Patterns or clusters of multimorbidity | Medical records and administrative data | Yes | Count of conditions (EDC identified by ACG) | Yes | 61 | Yes-explicit |
| Corrao, Argano et al. 2019 [23]  Corrao, Natoli et al. 2020 [24] | 4714 adults  80 (79.3-79.7) years  51% female | Internal medicine and geriatric medicine wards, University Hospital  [Inpatient]  Italy | Association of multimorbidity with an outcome  Patterns or clusters of multimorbidity  Performance of the measure, to itself or others, in characterising multimorbidity | Medical records and administrative data | No | Weighted index of body systems (CIRS-CI, CIRS-SI)  Count of conditions | Yes (CIRS)  No (count) | 14 body systems  Count not reported | Yes-explicit |
| Das, Bardakci et al. 2022 [25] | 1142 adults  87±2 years  56% female | ED admissions, University Hospital  [ED]  Turkey | Association of multimorbidity with an outcome | Medical records and administrative data | No | Weighted index of conditions (CCI) | Yes | 16 | No |
| Dattalo, DuGoff et al. 2017 [26] | 710,609 adults  Median 78 (72-85) years  63% female | National medicare claims data  [Inpatient]  USA | Performance of the measure, to itself or others, in characterising multimorbidity  Association of multimorbidity with an outcome | Medical records and administrative data | Yes | Counts of conditions (Medicare CCW Conditions, EDC within ACG, Medicare Advantage c-SNP, CCI conditions) | Yes | CCW=21 (19 for men, 21 for female)  EDC=119  cSNP=15  CCI=17 | Yes-implicit |
| Dias, Teixeira-Lopes et al. 2015 [27] | 100 adults  81±8 years  58% female | Acute internal medicine ward, University Hospital [Inpatient]  Portugal | Performance of the measure, to itself or others, in characterising multimorbidity  Association of multimorbidity with an outcome | Medical records and administrative data | No | Weighted index of conditions (CCI)  Weighted index of body systems (CIRS-G)  Weighted index of medications (MDBI) | Yes | CCI=19  CIRS-G=14  MDBI=20 | No |
| Díez-Manglano, Giménez-López et al. 2015 [28] | 457 adults  81±9 years  55% female | Internal medicine and acute geriatric ward, 13 hospitals  [Inpatient]  Spain | Study of populations with multimorbidity | Medical records and administrative data | Yes | Weighted index of conditions (CCI)  Count of conditions | Yes | Count of conditions=15  CCI=19 | Yes-implicit |
| Enríquez-Gómez, Ortega-Navarro et al. 2022 [29] | 201 adults  81 years (SD not reported)  54% female | ED attendances, Tertiary hospital  [ED]  Spain | Performance of the measure, to itself or others, in characterising multimorbidity | Medical records and administrative data | No | Weighted index of conditions (CCI) | Yes | Not reported | No |
| Félix and Henriques 2021 [30] | 245 adults  78±7 years  51% female | General medical ward, Teaching hospital [Inpatient]  Portugal | Study of populations with multimorbidity | Medical records and administrative data | Yes | Count of conditions | No | Not reported | No |
| Guido, Perna et al. 2015 [31] | 80 adults,  83±7 years  73% female | Geriatric rehabilitation hospital [Inpatient]  Italy | Association of multimorbidity with an outcome | Self-report | No | Weighted index of body systems (CIRS-G) | Yes | 14 body systems | No |
| Helvik, Engedal et al. 2013 [32] | 484 adults  81±7 years  50% female | Internal medical patients, Community Hospital [Inpatient]  Norway | Association of multimorbidity with an outcome | Medical records and administrative data | No | Weighted index of conditions (Quan-CCI) | Yes | 17 | Yes-implicit |
| Incalzi, Capparella et al. 1997 [33] | 370 adults,  79±6 years  55% female | Medical and geriatric wards, University hospital [Inpatient]  Italy | Association of multimorbidity with an outcome | Medical records and administrative data | No | Weighted index of conditions (comorbidity index and Age-comorbidity index) | Yes | 52 | No |
| Jungo, Streit et al. 2021 [34] | 653,121 adults with multimorbidity and polypharmacy from 2007-2014,  ~78±8 years  62% female | Medicare claims from seven secondary or tertiary care hospitals, Boston, Massachusetts [Inpatient and Outpatient]  USA | Study of populations with multimorbidity | Medical records and administrative data | Yes | Count of conditions | Yes | 77 | No |
| Juul-Larsen, Andersen et al. 2020 [35] | 369 adults  Median age 78 (IQR, 71-85)  62% female | Medical ED attendances  [ED]  Denmark | Association of multimorbidity with an outcome  Patterns or clusters of multimorbidity | Not reported | Yes | Count of conditions | Yes | 22 | Yes-implicit |
| Kabboord, Godfrey et al. 2020 [36] | 98 adults,  Median age 82 (IQR 11, 77-88),  61% female | Inpatient geriatric rehabilitation [Inpatient]  UK | Association of multimorbidity with an outcome  Patterns or clusters of multimorbidity | Medical records and administrative data | No | Weighted index of conditions (CCI, FCI, w-FCI) | Yes | CCI=19  FCI=18  w-FCI=18 | Yes-implicit |
| Kumar, Graham et al. 2016 [37] | 105,441 adults  79±7 years  64.3% female | Inpatient rehabilitation facility, Medicare claims data  [Inpatient]  USA | Association of multimorbidity with an outcome  Patterns or clusters of multimorbidity | Medical records and administrative data | No | Count of conditions (HCC)  Weighted index of conditions (Deyo-CCI, ECI, FCI, CMS-comorbidity tiers) | Yes | Deyo-CCI=17  ECI=30  FCI=18  HCC=79  CMS comorbidity tiers=4 | Yes implicit |
| Kutz, Koch et al. 2022 [38] | RCT of an electronic discharge planning tool in multimorbidity patients.  Control hospitals:  438,791 adults  74 years (60-83) [median, IQR]  49% female  Intervention Hospitals:  54,695 adults  72 years (59-82)  47% female | Acute medical admissions, 82 hospitals [Inpatient]  Switzerland | Study of populations with multimorbidity | Not reported | Yes | Weighted index of conditions (ECI) | No | Not reported | Yes-explicit |
| Lai, Huang et al. 2022 [39] | 144,567 adults  74±7 years  54% female | Inpatient and outpatient claims for Taiwan Healthcare [Inpatient and Outpatient]  Taiwan | Association of multimorbidity with an outcome | Medical records and administrative data | No | Weighted index of conditions (multimorbidity frailty index) | Yes | 38 | Yes-implicit |
| Lekan, McCoy et al. 2021 [40] | 55,778 adults  69±11 years  53% female | Hospital inpatients, 5 hospitals [Inpatient]  USA | Association of multimorbidity with an outcome  Performance of the measure, to itself or others, in characterising multimorbidity | Medical records and administrative data | Yes | Weighted index of conditions (CCI-12, CCI-17, ECI)  Multi-factorial index (FRS) | Yes | CCI-17=17  CCI-12=12  ECI=30  FRS=26 | Yes-explicit |
| Liang, Hann Lin et al. 2021 [41] | 200 adults  81±7 years  58% female | Hospital inpatients with high risk of readmission [Inpatient]  Taiwan | Study of populations with multimorbidity | Medical records and administrative data | No | Not reported | Not reported | Not reported | Yes-implicit |
| Lv, Li et al. 2022 [42] | 3836 adults  74±6 years  44% female | Internal medicine inpatients,  5 tertiary hospitals [Inpatient]  China | Study of populations with multimorbidity | Medical records and administrative data  Self-report | Yes | Count of conditions | Yes | 12 | No |
| Martínez-Velilla, Cambra-Contin et al. 2014 [43] | 122 adults  85±5 years  57% female | Acute geriatric ward,  Tertiary Hospital [Inpatient]  Spain | Association of multimorbidity with an outcome  Performance of the measure, to itself or others, in characterising multimorbidity | Self-report | Not reported | Weighted index of conditions, function, and laboratory measures (BISEP)  Weighted index of conditions (PI, CCI, GIC, ICED)  Weighted index of body systems (CIRS)  Count of conditions | Yes | BISEP=10  PI=not reported  CCI=19  GIC=15  ICED=19 medical conditions, 11 physical impairments  CIRS=14 body systems  Count of conditions=not reported | Yes-implicit |
| Mehta, Li et al. 2022 [44] | 899,844 adults  79±8 years  56% female | Medicare beneficiaries hospitalised at least once [Inpatient]  USA | Association of multimorbidity with an outcome | Medical records and administrative data | No | Weighted index of conditions (S-ECI, AHRQ-ECI, Quan-CCI) | Yes | Quan-CCI=17  AHRQ-ECI=38  S-ECI=38 | No |
| Müller, Huembelin et al. 2021 [45] | 1,463,781 adults  68 ± 17 years  49% female | Medical inpatients, Secondary and tertiary care hospitals [Inpatient]  Switzerland | Prevalence or burden of multimorbidity  Association of multimorbidity with an outcome | Medical records and administrative data | Yes | Count of conditions | Yes | 11 disease categories | Yes-implicit |
| Novella, Elli et al. 2021 [46] | REPOSI:  7505 adults  79 ± 8 years  52% female | Internal medicine and geriatric medicine wards [Inpatient]  Italy | Performance of the measure, to itself or others, in characterising multimorbidity | Medical records and administrative data | Yes | Weighted index of conditions (CCI)  Weighted index of medications (CDS, MCI DDCI) | Yes | CCI=23  CDS=17  MCI=20  DDCI=19 drug classes | Yes-explicit |
| Novella, Elli et al. 2022 [47] | REPOSI:  4765 adults  79±8 years  52% female  ELICADHE:  633 adults  91±6 years  58% female | REPOSI:  Internal medicine and geriatric medicine wards [Inpatient]  Italy  ELICADHE:  20 internal medicine and geriatric wards [Inpatient]  Italy | Association of multimorbidity with an outcome  Performance of the measure, to itself or others, in characterising multimorbidity | Medical records and administrative data | Yes | Weighted index of conditions (CCI)  Weighted index of medications (CDS, MCI DDCI) | Yes | CCI=23  CDS=17  MCI=20  DDCI=19 drug classes | No |
| Olsson, Terent et al. 2005 [48] | 885 adults  69±7 years  51% female | Nonsurgical inpatients, University Hospital [Inpatient]  Sweden | Association of multimorbidity with an outcome | Medical records and administrative data | No | Weighted index of conditions (CCI) | Yes | CCI=17 | No |
| Oterino-Moreira, Lorenzo-Martinez et al. 2022 [49] | 79,425 admissions  Median age 72 (IQR 56-80)  46% female | Inpatients, Public Hospital [Inpatient]  Spain | Association of multimorbidity with an outcome  Performance of the measure, to itself or others, in characterising multimorbidity | Medical records and administrative data | Yes | Weighted index of conditions (CCI, ECI, EVWCI) | Yes | CCI=17  ECI=30  EVWCI=30 | No |
| Patrick, Knoefel et al. 2001 [50] | 110 adults  83±7 years  71% female | Geriatric Rehabilitation Inpatient Unit [Inpatient]  Canada | Association of multimorbidity with an outcome | Medical records and administrative data | No | Weighted index of systems (CIRS) | Yes | CIRS=13 | No |
| Peng, Lin et al. 2014 [51] | 401 adults  85±6 years  26% female | Geriatric Admission Ward,  Taipei Veterans Hospital [Inpatient]  Taiwan | Association of multimorbidity with an outcome | Medical records and administrative data | No | Weighted index of conditions (CCI) | Yes | Not reported | No |
| Rosen, Roberts et al. 2022 [52] | 174,891 adults  73±12 years  54% female | Emergency general surgery patients [Inpatient]  USA | Association of multimorbidity with an outcome | Medical records and administrative data | Yes | Count of disease clusters (Silber-QCS) | Yes | Silber-QCS=113 | No |
| Rosen, Wirtalla et al. 2022 [53] | 312,160 adults  72±13 years  46% female | Emergency general surgery patients [Inpatient]  USA | Association of multimorbidity with an outcome | Medical records and administrative data | Yes | Count of disease clusters (QCS)  Weighted index of conditions (ECI) | Yes | QCS=113  ECI=31 | No |
| Rozzini, Frisoni et al. 2002 [54] | 493 adults  79±7 years  71% female | Inpatients, Geriatric Rehabilitation Hospital [Inpatient]  Italy | Performance of the measure, to itself or others, in characterising multimorbidity  Association of multimorbidity with an outcome | Medical records and administrative data | No | Count of conditions  Weighted index of conditions (GIC, Greenfield- IDS) | Yes | Count of conditions=15  Greenfield-IDS=15  GIC=15 | Yes-implicit |
| Sangha, Stucki et al. 2003 [55] | 170 adults  65±9 years  55% female | Medical and surgical inpatients, Brigham and Female’s Hospital [Inpatient]  USA | Association of multimorbidity with an outcome | Medical records and administrative data  Self-report | No | Weighted index of conditions (CCI)  Count of conditions (self-administered comorbidity questionnaire) | Yes | CCI=19  Self-administered comorbidity questionnaire =16 | Yes-implicit |
| Sara, Chowdhury et al. 2018 [56] | 566 adults  Age frequencies:  60-69 years:81%  70-79 years:13%  80+ years: 6%  24% female | Two tertiary hospitals [Inpatient]  Bangladesh | Prevalence or burden of multimorbidity | Medical records and administrative data | Yes | Weighted index of systems (CIRS-c) | Yes | CIRS=14 | Yes-explicit |
| Schaer, Patel et al. 2020[57] | 238 adults  75±8 years  57% female | Medical inpatients with moderate or severe pain [Inpatient]  USA | Study of populations with multimorbidity | Medical records and administrative data | Yes | Weighted index of body systems (CIRS-G) | Yes | CIRS-G=14 | Yes-explicit |
| Schneider, Aubert et al. 2022 [58] | 1879 adults,  Median age 79 (IQR 74-84)  44% female | Multimorbid hospital inpatients [Inpatient]  Belgium, Netherlands, Republic of Ireland, Switzerland | Study of populations with multimorbidity  Association of multimorbidity with an outcome | Medical records and administrative data | Yes | Weighted index of conditions (CCI, GI)  Weighted index of conditions, functional and laboratory measures (BISEP)  Weighted index of conditions, healthcare utilisation, and age (LI)  Weighted index of physical function, sex, conditions, and laboratory measures (WI) | Yes | CCI=19  GI=20  BISEP=10  LI=6  WI=2 | Yes-implicit |
| Shakib, Dundon et al. 2016 [59] | Multimorbid population for specialised outpatient follow-up after discharge from hospital.  Intervention group  252 adults  Median age 77 (IQR 65-82) years  50% female  Control group  1008 adults  Median age 77 (IQR 66-82) years  50% female | Outpatient clinic, Tertiary Hospital, [Outpatient]  Australia | Study of populations with multimorbidity | Medical records and administrative data | Yes | Count of conditions | Not reported | No | Yes-explicit |
| Shih, Gerrard et al. 2015 [60] | 120,957 adults  71±14 years  54% female | Inpatient rehabilitation hospitals [Inpatient]  USA | Association of multimorbidity with an outcome  Study of populations with multimorbidity | Medical records and administrative data | No | Weighted index of conditions (Deyo-CCI, ECI,  CMS-comorbidity tiers) | Yes | Deyo-CCI=17  ECI=29  CMS-comorbidity tiers=4 tiers | Yes-explicit |
| Shih, Zafonte et al. 2016 [61] | 4,199,002 adult admissions,  69±15 years  57% female | 1158 US inpatient rehabilitation facilities [Inpatient]  USA | Association of multimorbidity with an outcome | Medical records and administrative data | No | Count of conditions  Weighted index of conditions (Deyo-CCI, ECI, CMS- comorbidity tier system) | Yes-for indices  No-for count | Count=not reported  Deyo-CCI=17  ECI=29  CMS-comorbidity tiers=4 tiers | Yes-explicit |
| Silber, Rosenbaum et al. 2023 [62] | 30,958 adults  74 years (SD not reported)  35.1% female | Surgical patients, Ambulatory Surgical Centres [Outpatient]  USA | Study of populations with multimorbidity | Medical records and administrative data | Yes | Count of disease clusters (Silber-QCS) | Yes | Silber QCS=113 | No |
| Susser, McCusker et al. 2008 [63] | 520 adults  100% of population aged 65+, no measure of average reported.  60% female | ED patients, 4 hospitals in Montreal  [ED]  Canada | Performance of the measure, to itself or others, in characterising multimorbidity  Association of multimorbidity with an outcome | Self-report  Medical records and administrative data | No | Weighted index of conditions (CCI) | Yes | CCI=18 | No |
| Tan, Ng et al. 2014 [64] | 448 adults  68.6±71.2 years  42.6% female | Internal medicine patients, General Hospital [Inpatient]  Singapore | Association of multimorbidity with an outcome | Not reported | No | Weighted index of conditions (CCI) | Yes | CCI=19 | No |
| Thanakiattiwibun, Siriussawakul et al. 2023 [65] | 360 adults  74±7 years  62% female | Surgical patients, University Hospital [Inpatient]  Thailand | Prevalence or burden of multimorbidity  Association of multimorbidity with an outcome | Medical records and administrative data | Yes | Count of conditions  Weighted index of conditions (CCI) | No | CCI=16  Count=16 | Yes-implicit |
| Ünlü, Geyīk et al. 2022 [66] | 300 adults  Median 71 years (range 65-95 years)  47% female | Elective surgery patients, University Hospital [Outpatient]  Turkey | Performance of the measure, to itself or others, in characterising multimorbidity  Association of multimorbidity with an outcome | Medical records and administrative data | No | Weighted index of conditions (CCI-A, mFI-5) | Yes | CCI-A=18  mFI-5=5 | No |
| Vu, Nguyen et al. 2019 [67] | 405 adults  72±9 years  60% female | Outpatient attendances and inpatients [Outpatient and Inpatient]  Vietnam | Association of multimorbidity with an outcome  Prevalence or burden of multimorbidity | Medical records and administrative data | Yes | Count of conditions | No | Not reported | Yes-implicit |
| Wei 2023 [68]  Wei and Cho 2023 [69] | 10,737 adults  76±9 years  57% female | Medicare Parts A and B linked to the Health and Retirement Study [Inpatient]  USA | Association of multimorbidity with an outcome | Medical records and administrative data | No | Weighted index of conditions (CCI, ECI, MWI) | Indices described  Count not described | MWI=84  CCI=19  ECI=30  Disease count | Yes-implicit |
| Williams, Griffies et al. 2021 [70] | 551 adults.  Cohort demographics split into survived and deceased.  Survived  -533 adults  -Median age 65 (IQR, 47-78) years  -40% female  Deceased  -18 adults  -Median age 81 (IQR, 60-89) years  -50% female | Suspected infection patients, Emergency Department, Tertiary Hospital  [ED]  Australia | Association of multimorbidity with an outcome | Medical records and administrative data | No | Weighted index of conditions (CCI) | No | 19 | No |
| Zekry, Loures Valle et al. 2010 [71]  Zekry, Loures Valle et al. 2012 [72] | 444 adults  85±7 years  74% female | Acute geriatric hospital, Geneva  [Inpatient]  Switzerland | Performance of the measure, to itself or others, in characterising multimorbidity  Association of multimorbidity with an outcome | Medical records and administrative data | No | Weighted index of body systems (CIRS-G)  Weighted index of conditions (CCI, GIC, ICED, KS)  Weighted index of medications (CDS) | Yes | CIRS-G=14  CCI=19  GIC=15  ICED=15 conditions, 12 physical impairment  KS=14  CDS=30 | Yes-implicit |
| Zhang, Ma et al. 2020 [73] | 4,348 adults  74±8 years  38% female | Geriatric medicine departments,  12 tertiary hospitals [Inpatient]  China | Prevalence or burden of multimorbidity  Risk factors for developing multimorbidity | Self-report | Yes | Count of conditions | No | 15 | No |
| Zhang, Wu et al. 2022 [74] | 3,911 adults  73±6 years  51% female | 25 general hospitals  [Inpatient]  China | Association of multimorbidity with an outcome | Medical records and administrative data | No | Weighted index of conditions (CCI-A) | Yes | 17 | No |
| Zhang, Yang et al. 2021 [75] | 5,759 adults  75±8 years  51% female | Internal medicine, surgery, geriatric, and ICU departments,  Wuhan hospital [Inpatient]  China | Association of multimorbidity with an outcome | Medical records and administrative data | Yes | Weighted index of conditions (CCI-A) | Yes | 17 | No |

ACG=adjusted clinical group; BISEP (Burden of Illness Score for Elderly Persons); CCW=Chronic Condition Warehouse; CCI= Charlson Comorbidity Index; CCI-A=Age adjusted CCI; DCCI= Deyo Charlson Comorbidity Index; MDBI=Medication-Based Disease Burden Index; CDS=Chronic Disease Score; CPS=comorbidity polypharmacy score; CIRS-CI=Cumulative Illness Rating Scale Comorbidity Index; CIRS-SI=Cumulative Illness Rating Scale Severity Index; CIRS-G=Cumulative Illness Rating Scale for Geriatrics; CRG= clinical risk groups; CMS= Centres for Medicare and Medicaid Services; DDCI=Drug-derived Comorbidity Index; ECI= Elixhauser Comorbidity Index; S-ECI=Summary Elixhauser Comorbidity Index; AHRQ-ECI=Agency for Healthcare Research and Quality Elixhauser Comorbidity Index; EVWCI=Elixhauser- Van Walraven Comorbidity Index; EDC=Expanded disease clusters; FCI=Functional Comorbidity Index; w-FCI=weighted functional comorbidity index; FRS=frailty risk score; GI= Gagné Index; GIC=Geriatric Index of Comorbidity; Greenfield-IDS=Greenfield Individual Disease Severity; HCC=Hierarchical Condition Category; ICED=Index of Coexistent Disease; KS=Kaplan Scale; LI=Levine Index; MCI=Medicine Comorbidity Index; MWI=Multimorbidity-weighted index; PI=Prognostic Index; QCS=qualifying comorbidity set; c-SNP=Special Needs Plan Chronic Conditions; WI=Walter Index

References

1. Amasene M, et al. Malnutrition and Poor Physical Function Are Associated With Higher Comorbidity Index in Hospitalized Older Adults. Front Nutr. 2022. **9**: p. 920485 DOI: <https://doi.org/10.3389/fnut.2022.920485>.

2. Arfken CL, Lichtenberg PA, and Kuiken T. Special Feature: Importance of Comorbid Illnesses in Predicting Mortality for Geriatric Rehabilitation. Topics in Geriatric Rehabilitation. 1998. **13**(4): p. 69-76 DOI: <https://doi.org/10.1097/00013614-199806000-00009>.

3. Aubert CE, et al. Multimorbidity and healthcare resource utilization in Switzerland: a multicentre cohort study. BMC Health Services Research. 2019. **19**(1): p. 708 DOI: <https://doi.org/10.1186/s12913-019-4575-2>.

4. Bahat G, et al. Assessments of functional status, comorbidities, polypharmacy, nutritional status and sarcopenia in Turkish community-dwelling male elderly. Aging Male. 2013. **16**(2): p. 67-72 DOI: <https://doi.org/10.3109/13685538.2013.771329>.

5. Bahat G, et al. Comorbidities, polypharmacy, functionality and nutritional status in Turkish community-dwelling female elderly. Aging Clin Exp Res. 2014. **26**(3): p. 255-9 DOI: <https://doi.org/10.1007/s40520-014-0229-8>.

6. Bahrmann A, et al. The Charlson Comorbidity and Barthel Index predict length of hospital stay, mortality, cardiovascular mortality and rehospitalization in unselected older patients admitted to the emergency department. Aging Clin Exp Res. 2019. **31**(9): p. 1233-1242 DOI: <https://doi.org/10.1007/s40520-018-1067-x>.

7. Bayliss EA, et al. Effect of continuity of care on hospital utilization for seniors with multiple medical conditions in an integrated health care system. Ann Fam Med. 2015. **13**(2): p. 123-9 DOI: <https://doi.org/10.1370/afm.1739>.

8. Beloosesky Y, Weiss A, and Mansur N. Validity of the Medication-based Disease Burden Index compared with the Charlson Comorbidity Index and the Cumulative Illness Rating Scale for geriatrics: a cohort study. Drugs Aging. 2011. **28**(12): p. 1007-14 DOI: <https://doi.org/10.2165/11597040-000000000-00000>.

9. Bernard S, Inderjeeth C, and Raymond W. Higher Charlson Comorbidity Index scores do not influence Functional Independence Measure score gains in older rehabilitation patients. Australas J Ageing. 2016. **35**(4): p. 236-241 DOI: <https://doi.org/10.1111/ajag.12351>.

10. Briongos-Figuero LS, et al. Evaluation and characterization of multimorbidity profiles, resource consumption and healthcare needs in extremely elderly people. Int J Qual Health Care. 2020. **32**(4): p. 266-270 DOI: <https://doi.org/10.1093/intqhc/mzaa022>.

11. Buurman BM, et al. Acute and chronic diseases as part of multimorbidity in acutely hospitalized older patients. Eur J Intern Med. 2016. **27**: p. 68-75 DOI: <https://doi.org/10.1016/j.ejim.2015.09.021>.

12. Canaslan K, et al. Predictivity of the comorbidity indices for geriatric syndromes. BMC Geriatr. 2022. **22**(1): p. 440 DOI: <https://doi.org/10.1186/s12877-022-03066-8>.

13. Canevelli M, et al. Counting deficits or diseases? The agreement between frailty and multimorbidity in subjects with cognitive disturbances. Aging Clin Exp Res. 2020. **32**(1): p. 179-182 DOI: <https://doi.org/10.1007/s40520-019-01161-2>.

14. Capisizu A, et al. Findings regarding the relationships between sociodemographic, psychological, comorbidity factors, and functional status, in geriatric inpatients. Adv Exp Med Biol. 2015. **821**: p. 45-55 DOI: <https://doi.org/10.1007/978-3-319-08939-3_9>.

15. Chan CH, et al. The association of comorbidity measures and mortality in geriatric rehabilitation inpatients by cancer status: RESORT. Support Care Cancer. 2021. **29**(8): p. 4513-4519 DOI: <https://doi.org/10.1007/s00520-020-05967-z>.

16. Chan TC, et al. Validation study of Charlson Comorbidity Index in predicting mortality in Chinese older adults. Geriatr Gerontol Int. 2014. **14**(2): p. 452-7 DOI: <https://doi.org/10.1111/ggi.12129>.

17. Chen H, Chen Y, and Cui B. The association of multimorbidity with healthcare expenditure among the elderly patients in Beijing, China. Arch Gerontol Geriatr. 2018. **79**: p. 32-38 DOI: <https://doi.org/10.1016/j.archger.2018.07.008>.

18. Cheng C, Inder K, and Chan SW. The relationship between coping strategies and psychological distress in Chinese older adults with multiple chronic conditions. Australas J Ageing. 2021. **40**(4): p. 397-405 DOI: <https://doi.org/10.1111/ajag.12946>.

19. Chou HC, Huang CT, and Sheng WH. Differential roles of comorbidity burden and functional status in elderly and non-elderly patients with infections in general wards. J Formos Med Assoc. 2020. **119**(4): p. 821-828 DOI: <https://doi.org/10.1016/j.jfma.2019.08.032>.

20. Chow SK and Wong FK. A randomized controlled trial of a nurse-led case management programme for hospital-discharged older adults with co-morbidities. J Adv Nurs. 2014. **70**(10): p. 2257-71 DOI: <https://doi.org/10.1111/jan.12375>.

21. Chua MT, et al. Comparing Comorbidity Polypharmacy Score and Charlson Comorbidity Index in predicting outcomes in older trauma patients. Injury. 2023. **54**(4): p. 1113-1118 DOI: <https://doi.org/10.1016/j.injury.2023.02.031>.

22. Clerencia-Sierra M, et al. Multimorbidity Patterns in Hospitalized Older Patients: Associations among Chronic Diseases and Geriatric Syndromes. PLoS One. 2015. **10**(7): p. e0132909 DOI: <https://doi.org/10.1371/journal.pone.0132909>.

23. Corrao S, et al. Sex-Differences in the Pattern of Comorbidities, Functional Independence, and Mortality in Elderly Inpatients: Evidence from the RePoSI Register. J Clin Med. 2019. **8**(1) DOI: <https://doi.org/10.3390/jcm8010081>.

24. Corrao S, et al. Comorbidity does not mean clinical complexity: evidence from the RePoSI register. Intern Emerg Med. 2020. **15**(4): p. 621-628 DOI: <https://doi.org/10.1007/s11739-019-02211-3>.

25. Das M, et al. Prediction of mortality with Charlson Comorbidity Index in super-elderly patients admitted to a tertiary referral hospital. Çukurova Araştırmaları Dergisi. 2022. **47**: p. 199-207 DOI: <https://doi.org/10.17826/cumj.1017164>.

26. Dattalo M, et al. Apples and Oranges: Four Definitions of Multiple Chronic Conditions and their Relationship to 30-Day Hospital Readmission. J Am Geriatr Soc. 2017. **65**(4): p. 712-720 DOI: <https://doi.org/10.1111/jgs.14539>.

27. Dias A, et al. Comorbidity burden assessment in older people admitted to a Portuguese University Hospital. Aging Clin Exp Res. 2015. **27**(3): p. 323-8 DOI: <https://doi.org/10.1007/s40520-014-0280-5>.

28. Díez-Manglano J, et al. Excessive polypharmacy and survival in polypathological patients. Eur J Clin Pharmacol. 2015. **71**(6): p. 733-739 DOI: <https://doi.org/10.1007/s00228-015-1837-8>.

29. Enríquez-Gómez A, et al. Comparison of a polypharmacy-based scale with Charlson comorbidity index to predict 6-month mortality in chronic complex patients after an ED visit. Br J Clin Pharmacol. 2022. **88**(4): p. 1795-1803 DOI: <https://doi.org/10.1111/bcp.15096>.

30. Félix IB and Henriques A. Medication adherence and related determinants in older people with multimorbidity: A cross-sectional study. Nurs Forum. 2021. **56**(4): p. 834-843 DOI: <https://doi.org/10.1111/nuf.12619>.

31. Guido D, et al. A comorbidity prognostic effect on post-hospitalization outcome in a geriatric rehabilitation setting: the pivotal role of functionality, assessed by mediation model, and association with the Brass index. Aging Clin Exp Res. 2015. **27**(6): p. 849-56 DOI: <https://doi.org/10.1007/s40520-015-0360-1>.

32. Helvik AS, Engedal K, and Selbæk G. Three-year mortality in previously hospitalized older patients from rural areas--the importance of co-morbidity and self-reported poor health. BMC Geriatr. 2013. **13**: p. 17 DOI: <https://doi.org/10.1186/1471-2318-13-17>.

33. Incalzi RA, et al. The interaction between age and comorbidity contributes to predicting the mortality of geriatric patients in the acute-care hospital. J Intern Med. 1997. **242**(4): p. 291-8 DOI: <https://doi.org/10.1046/j.1365-2796.1997.00132.x>.

34. Jungo KT, Streit S, and Lauffenburger JC. Utilization and Spending on Potentially Inappropriate Medications by US Older Adults with Multiple Chronic Conditions using Multiple Medications. Archives of Gerontology and Geriatrics. 2021. **93**: p. 104326 DOI: <https://doi.org/10.1016/j.archger.2020.104326>.

35. Juul-Larsen HG, et al. Differences in function and recovery profiles between patterns of multimorbidity among older medical patients the first year after an acute admission-An exploratory latent class analysis. Arch Gerontol Geriatr. 2020. **86**: p. 103956 DOI: <https://doi.org/10.1016/j.archger.2019.103956>.

36. Kabboord AD, et al. The modified functional comorbidity index performed better than the Charlson index and original functional comorbidity index in predicting functional outcome in geriatric rehabilitation: a prospective observational study. BMC Geriatr. 2020. **20**(1): p. 114 DOI: <https://doi.org/10.1186/s12877-020-1498-z>.

37. Kumar A, et al. Examining the Association Between Comorbidity Indexes and Functional Status in Hospitalized Medicare Fee-for-Service Beneficiaries. Phys Ther. 2016. **96**(2): p. 232-40 DOI: <https://doi.org/10.2522/ptj.20150039>.

38. Kutz A, et al. Association of Interprofessional Discharge Planning Using an Electronic Health Record Tool With Hospital Length of Stay Among Patients with Multimorbidity: A Nonrandomized Controlled Trial. JAMA Netw Open. 2022. **5**(9): p. e2233667 DOI: <https://doi.org/10.1001/jamanetworkopen.2022.33667>.

39. Lai HY, et al. Development of frailty index using ICD-10 codes to predict mortality and rehospitalization of older adults: An update of the multimorbidity frailty index. Arch Gerontol Geriatr. 2022. **100**: p. 104646 DOI: <https://doi.org/10.1016/j.archger.2022.104646>.

40. Lekan DA, et al. Comparison of a Frailty Risk Score and Comorbidity Indices for Hospital Readmission Using Electronic Health Record Data. Res Gerontol Nurs. 2021. **14**(2): p. 91-103 DOI: <https://doi.org/10.3928/19404921-20210115-03>.

41. Liang HY, et al. Effectiveness of a Nurse-Led Tele-Homecare Program for Patients With Multiple Chronic Illnesses and a High Risk for Readmission: A Randomized Controlled Trial. J Nurs Scholarsh. 2021. **53**(2): p. 161-170 DOI: <https://doi.org/10.1111/jnu.12622>.

42. Lv J, et al. Research on the frailty status and adverse outcomes of elderly patients with multimorbidity. BMC Geriatr. 2022. **22**(1): p. 560 DOI: <https://doi.org/10.1186/s12877-022-03194-1>.

43. Martínez-Velilla N, Cambra-Contin K, and Ibáñez-Beroiz B. Comorbidity and prognostic indices do not improve the 5-year mortality prediction of components of comprehensive geriatric assessment in hospitalized older patients. BMC Geriatr. 2014. **14**: p. 64 DOI: <https://doi.org/10.1186/1471-2318-14-64>.

44. Mehta HB, et al. Development and Validation of the Summary Elixhauser Comorbidity Score for Use With ICD-10-CM-Coded Data Among Older Adults. Ann Intern Med. 2022. **175**(10): p. 1423-1430 DOI: <https://doi.org/10.7326/m21-4204>.

45. Müller M, et al. Association of in-hospital multimorbidity with healthcare outcomes in Swiss medical inpatients. Swiss Med Wkly. 2021. **151**: p. w20405 DOI: <https://doi.org/10.4414/smw.2021.20405>.

46. Novella A, et al. Comparison between drug therapy-based comorbidity indices and the Charlson Comorbidity Index for the detection of severe multimorbidity in older subjects. Aging Clin Exp Res. 2021. **33**(7): p. 1929-1935 DOI: <https://doi.org/10.1007/s40520-020-01706-w>.

47. Novella A, et al. Relation between drug therapy-based comorbidity indices, Charlson's comorbidity index, polypharmacy and mortality in three samples of older adults. Arch Gerontol Geriatr. 2022. **100**: p. 104649 DOI: <https://doi.org/10.1016/j.archger.2022.104649>.

48. Olsson T, Terent A, and Lind L. Charlson Comorbidity Index can add prognostic information to Rapid Emergency Medicine Score as a predictor of long-term mortality. Eur J Emerg Med. 2005. **12**(5): p. 220-4 DOI: <https://doi.org/10.1097/00063110-200510000-00004>.

49. Oterino-Moreira I, et al. Comparison of Three Comorbidity Measures for Predicting In-Hospital Death through a Clinical Administrative Nacional Database. Int J Environ Res Public Health. 2022. **19**(18) DOI: <https://doi.org/10.3390/ijerph191811262>.

50. Patrick L, et al. Medical comorbidity and rehabilitation efficiency in geriatric inpatients. J Am Geriatr Soc. 2001. **49**(11): p. 1471-7 DOI: <https://doi.org/10.1046/j.1532-5415.2001.4911239.x>.

51. Peng LN, et al. Nutritional status plays the mediating role of the functional status and comorbidity among older patients admitted to the Geriatric Evaluation and Management Unit: A Tobit model application. European Geriatric Medicine. 2014. **5**(2): p. 87-91 DOI: <https://doi.org/10.1016/j.eurger.2013.12.003>.

52. Rosen CB, et al. Analyzing Impact of Multimorbidity on Long-Term Outcomes after Emergency General Surgery: A Retrospective Observational Cohort Study. J Am Coll Surg. 2022. **235**(5): p. 724-735 DOI: <https://doi.org/10.1097/xcs.0000000000000303>.

53. Rosen CB, et al. Multimorbidity Confers Greater Risk for Older Patients in Emergency General Surgery Than the Presence of Multiple Comorbidities: A Retrospective Observational Study. Med Care. 2022. **60**(8): p. 616-622 DOI: <https://doi.org/10.1097/mlr.0000000000001733>.

54. Rozzini R, et al. Geriatric Index of Comorbidity: validation and comparison with other measures of comorbidity. Age Ageing. 2002. **31**(4): p. 277-85 DOI: <https://doi.org/10.1093/ageing/31.4.277>.

55. Sangha O, et al. The Self-Administered Comorbidity Questionnaire: a new method to assess comorbidity for clinical and health services research. Arthritis Rheum. 2003. **49**(2): p. 156-63 DOI: <https://doi.org/10.1002/art.10993>.

56. Sara HH, Chowdhury MAB, and Haque MA. Multimorbidity among elderly in Bangladesh. Aging Med (Milton). 2018. **1**(3): p. 267-275 DOI: <https://doi.org/10.1002/agm2.12047>.

57. Schear S, et al. Multimorbidity and Opioid Prescribing in Hospitalized Older Adults. J Palliat Med. 2020. **23**(4): p. 475-482 DOI: <https://doi.org/10.1089/jpm.2019.0260>.

58. Schneider C, et al. Comparison of 6 Mortality Risk Scores for Prediction of 1-Year Mortality Risk in Older Adults With Multimorbidity. JAMA Netw Open. 2022. **5**(7): p. e2223911 DOI: <https://doi.org/10.1001/jamanetworkopen.2022.23911>.

59. Shakib S, et al. Effect of a Multidisciplinary Outpatient Model of Care on Health Outcomes in Older Patients with Multimorbidity: A Retrospective Case Control Study. PLoS One. 2016. **11**(8): p. e0161382 DOI: <https://doi.org/10.1371/journal.pone.0161382>.

60. Shih SL, et al. Functional Status Outperforms Comorbidities in Predicting Acute Care Readmissions in Medically Complex Patients. J Gen Intern Med. 2015. **30**(11): p. 1688-95 DOI: <https://doi.org/10.1007/s11606-015-3350-2>.

61. Shih SL, et al. Functional Status Outperforms Comorbidities as a Predictor of 30-Day Acute Care Readmissions in the Inpatient Rehabilitation Population. J Am Med Dir Assoc. 2016. **17**(10): p. 921-6 DOI: <https://doi.org/10.1016/j.jamda.2016.06.003>.

62. Silber JH, et al. The Safety of Performing Surgery at Ambulatory Surgery Centers Versus Hospital Outpatient Departments in Older Patients With or Without Multimorbidity. Med Care. 2023. **61**(5): p. 328-337 DOI: <https://doi.org/10.1097/mlr.0000000000001836>.

63. Susser SR, McCusker J, and Belzile E. Comorbidity information in older patients at an emergency visit: self-report vs. administrative data had poor agreement but similar predictive validity. J Clin Epidemiol. 2008. **61**(5): p. 511-5 DOI: <https://doi.org/10.1016/j.jclinepi.2007.07.009>.

64. Tan C, et al. Disability impacts length of stay in general internal medicine patients. J Gen Intern Med. 2014. **29**(6): p. 885-90 DOI: <https://doi.org/10.1007/s11606-014-2815-z>.

65. Thanakiattiwibun C, et al. Multimorbidity, healthcare utilization, and quality of life for older patients undergoing surgery: A prospective study. Medicine (Baltimore). 2023. **102**(13): p. e33389 DOI: <https://doi.org/10.1097/md.0000000000033389>.

66. Ünlü EH, et al. Comparison of the Modified 5-item Frailty Index with the American Society of Anaesthesiologists Classification and Charlson Age Comorbidity Index for predicting postoperative outcomes in geriatric patients: A Prospective Observational Study. Turkish Journal of Geriatrics. 2022. **25**(4) DOI: <https://doi.org/10.31086/tjgeri.2022.320>.

67. Vu HM, et al. Effects of Chronic Comorbidities on the Health-Related Quality of Life among Older Patients after Falls in Vietnamese Hospitals. Int J Environ Res Public Health. 2019. **16**(19) DOI: <https://doi.org/10.3390/ijerph16193623>.

68. Wei MY. Multimorbidity, 30-Day Readmissions, and Postdischarge Mortality Among Medicare Beneficiaries Using a New ICD-Coded Multimorbidity-Weighted Index. J Gerontol A Biol Sci Med Sci. 2023. **78**(4): p. 727-734 DOI: <https://doi.org/10.1093/gerona/glac242>.

69. Wei MY and Cho J. Readmissions and postdischarge mortality by race and ethnicity among Medicare beneficiaries with multimorbidity. J Am Geriatr Soc. 2023. **71**(6): p. 1749-1758 DOI: <https://doi.org/10.1111/jgs.18251>.

70. Williams A, et al. Effect of age and comorbidity on the ability of quick-Sequential Organ Failure Assessment score to predict outcome in emergency department patients with suspected infection. Emerg Med Australas. 2021. **33**(4): p. 679-684 DOI: <https://doi.org/10.1111/1742-6723.13703>.

71. Zekry D, et al. Geriatrics index of comorbidity was the most accurate predictor of death in geriatric hospital among six comorbidity scores. J Clin Epidemiol. 2010. **63**(9): p. 1036-44 DOI: <https://doi.org/10.1016/j.jclinepi.2009.11.013>.

72. Zekry D, et al. Prospective comparison of 6 comorbidity indices as predictors of 1-year post-hospital discharge institutionalization, readmission, and mortality in elderly individuals. J Am Med Dir Assoc. 2012. **13**(3): p. 272-8 DOI: <https://doi.org/10.1016/j.jamda.2010.11.011>.

73. Zhang L, et al. A Multicenter Study of Multimorbidity in Older Adult Inpatients in China. J Nutr Health Aging. 2020. **24**(3): p. 269-276 DOI: <https://doi.org/10.1007/s12603-020-1311-x>.

74. Zhang XM, et al. Effect of the Age-Adjusted Charlson Comorbidity Index on All-Cause Mortality and Readmission in Older Surgical Patients: A National Multicenter, Prospective Cohort Study. Front Med (Lausanne). 2022. **9**: p. 896451 DOI: <https://doi.org/10.3389/fmed.2022.896451>.

75. Zhang Z, Yang H, and Luo M. Association Between Charlson Comorbidity Index and Community-Acquired Pressure Injury in Older Acute Inpatients in a Chinese Tertiary Hospital. Clin Interv Aging. 2021. **16**: p. 1987-1995 DOI: <https://doi.org/10.2147/cia.s338967>.
