# Supplementary material for: Approaches to characterising multimorbidity in older people accessing hospital care: a scoping review
Source: Eur Geriatr Med. 2025 Mar 1;16(4):1099–113. doi: 10.1007/s41999-025-01166-3 (PMC12378491; doi:10.1007/s41999-025-01166-3)
Supplement: Supplementary file 6 — Supplementary file6 (DOCX 263 KB) [file 41999_2025_1166_MOESM6_ESM.docx]

**Approaches to characterising multimorbidity in older people accessing hospital care**: **a scoping review**

Jonathan G Bunn^1,2^, Lewis Steell^1,2^, Susan J Hillman^1,2^, Miles D Witham^1,2^, Avan A Sayer^1,2^ and Rachel Cooper^1,2^ on behalf of the ADMISSION research collaborative

1. AGE Research Group, Translational and Clinical Research Institute, Faculty of Medical Sciences, Newcastle University, Newcastle upon Tyne, UK

2. NIHR Newcastle Biomedical Research Centre, Newcastle upon Tyne Hospitals NHS Foundation Trust, Cumbria Northumberland Tyne and Wear NHS Foundation Trust and Faculty of Medical Sciences, Newcastle University, Newcastle upon Tyne, UK

**Corresponding Author:**

Rachel Cooper, email rachel.cooper@newcastle.ac.uk

ORCID ID: 0000-0003-3370-5720

**Journal of Submission**: European Geriatric Medicine

Supplementary Information 6: Analyses of country of origin and weighted indices within included studies

Supplementary Information 6 Table 1: Summary of country of origin of included studies ordered by number of papers

| Country | Number of populations | Number of papers | Reference numbers of papers |
| --- | --- | --- | --- |
| USA | 15 (21%) | 16 (21%) | [1-16] |
| China^a^ | 8 (11%) | 8 (10%) | [17-24] |
| Italy | 7 (10%) | 8 (10%) | [25-32] |
| Spain | 7 (10%) | 7 (9%) | [33-39] |
| Switzerland | 5 (7%) | 6 (8%) | [40-45] |
| Turkey^a^ | 5 (7%) | 5 (6%) | [46-50] |
| Australia | 4 (6%) | 4 (5%) | [51-54] |
| Taiwan | 4 (6%) | 4 (5%) | [55-58] |
| Canada | 2 (3%) | 2 (3%) | [59, 60] |
| Netherlands | 2 (3%) | 2 (3%) | [43, 61] |
| Portugal | 2 (3%) | 2 (3%) | [62, 63] |
| Singapore | 2 (3%) | 2 (3%) | [64, 65] |
| Bangladesh^a^ | 1 (1%) | 1 (1%) | [66] |
| Belgium | 1 (1%) | 1 (1%) | [43] |
| Denmark | 1 (1%) | 1 (1%) | [67] |
| Germany | 1 (1%) | 1 (1%) | [68] |
| Israel | 1 (1%) | 1 (1%) | [69] |
| Norway | 1 (1%) | 1 (1%) | [70] |
| Romania | 1 (1%) | 1 (1%) | [71] |
| Republic of Ireland | 1 (1%) | 1 (1%) | [43] |
| Sweden | 1 (1%) | 1 (1%) | [72] |
| Thailand^a^ | 1 (1%) | 1 (1%) | [73] |
| UK | 1 (1%) | 1 (1%) | [74] |
| Vietnam^a^ | 1 (1%) | 1 (1%) | [75] |

Lower and middle income countries, as defined by the Organisation for Economic Co-operation and Development are highlighted by (^a^).

| Index | Number of study populations (n=72) | Reference number of papers |
| --- | --- | --- |
| Weighted index of conditions | 46 (64%) | [1-3, 5-8, 10, 12, 13, 16, 17, 22, 23, 29-34, 36-41, 43-45, 48-52, 54-56, 58, 60, 62, 64, 65, 68-70, 73, 74] |
| Weighted index of body systems | 9 (13%) | [11, 26-28, 38, 52, 59, 62, 66, 69] |
| Weighted index of medications | 7 (10%) | [30, 31, 44, 45, 48, 62, 64, 69] |
| Multifactorial indices | 3 (4%) | [34, 38, 43] |
| Count of disease clusters | 3 (4%) | [8, 9, 14] |

Supplementary Information 6 Table 2: Summary of weighted indices used across study populations within the scoping review

Supplementary Information 6 Table 3: Summary of the heterogeneity in the use of the Charlson Comorbidity Index (CCI) across studies ordered by version of CCI used, number of conditions and the use of grouping

| Version of CCI | Number of conditions | Grouping undertaken | Grouping details | Reference number of papers |
| --- | --- | --- | --- | --- |
| CCI | 14 | Yes | 0; 1; 2; 3; 4; ≥5 | [17] |
| CCI | 16 | Yes | 0; 1-2; 3-6; ≥7 | [64] |
| CCI | 16 | Yes | <6; ≥6 | [49] |
| CCI | 16 | No | - | [73] |
| CCI | 17 | Yes | 0-1; 2; 3 | [1] |
| CCI | 17 | No | - | [3] |
| CCI | 17 | No | - | [72] |
| CCI | 17 | No | - | [39] |
| CCI | 18 | Yes | 0; 1; 2; ≥3 | [60] |
| CCI | 18 | No | - | [65] |
| CCI | 19 | Yes | 0-2; 3-4; ≥5 | [68] |
| CCI | 19 | Yes | ≤4; 5-6; ≥7 | [51] |
| CCI | 19 | Yes | 0-1; 2; ≥3 | [34] |
| CCI | 19 | Yes | 0-4; ≥5 | [48] |
| CCI | 19 | Yes | 0-4; ≥5 | [62] |
| CCI | 19 | Yes | Quartiles | [38] |
| CCI | 19 | Yes | 0; 1-2; 3-4; ≥5 | [43] |
| CCI | 19 | Yes | Quartiles | [44, 45] |
| CCI | 19 | No | - | [69] |
| CCI | 19 | No | - | [52] |
| CCI | 19 | No | - | [36] |
| CCI | 19 | No | - | [74] |
| CCI | 19 | No | - | [10] |
| CCI | 19 | No | - | [16] |
| CCI | 19 | No | - | [54] |
| CCI | 23 | Yes | 0; 1-2; 3-4; ≥5 | [30] |
| CCI | 23 | Yes | 0; 1-2; 3-4; ≥5 | [31] |
| CCI | Not reported | Yes | ≤2; >2 | [55] |
| CCI | Not reported | Yes | 0-1; 2-3; ≥4 | [37] |
| CCI | Not reported | Yes | 0; 1-2; 3-4; ≥5 | [58] |
| Age-adjusted CCI (CCI-A) | 17 | Yes | 1-2; 3-4; ≥5 | [33] |
| CCI-A | 17 | Yes | <5; ≥5 | [23] |
| CCI-A | 17 | Yes | 2-3; 4-6; ≥7 | [22] |
| CCI-A | 18 | Yes | <5; ≥5 | [50] |
| CCI-A | 19 | No | - | [52] |
| Deyo-CCI | 17 | No | - | [5] |
| Deyo-CCI | 17 | No | - | [13] |
| Deyo-CCI | 17 | No | - | [12] |
| Deyo-CCI | Not reported | No | - | [40] |
| Quan-CCI | 17 | No | - | [70] |
| Quan-CCI | 17 | No | - | [7] |
| CCI-17 | 17 | No | - | [6] |
| CCI-12 | 12 | No | - | [6] |

References

1. Arfken CL, Lichtenberg PA, and Kuiken T. Special Feature: Importance of Comorbid Illnesses in Predicting Mortality for Geriatric Rehabilitation. Topics in Geriatric Rehabilitation. 1998. **13**(4): p. 69-76 DOI: <https://doi.org/10.1097/00013614-199806000-00009>.

2. Bayliss EA, et al. Effect of continuity of care on hospital utilization for seniors with multiple medical conditions in an integrated health care system. Ann Fam Med. 2015. **13**(2): p. 123-9 DOI: <https://doi.org/10.1370/afm.1739>.

3. Dattalo M, et al. Apples and Oranges: Four Definitions of Multiple Chronic Conditions and their Relationship to 30-Day Hospital Readmission. J Am Geriatr Soc. 2017. **65**(4): p. 712-720 DOI: <https://doi.org/10.1111/jgs.14539>.

4. Jungo KT, Streit S, and Lauffenburger JC. Utilization and Spending on Potentially Inappropriate Medications by US Older Adults with Multiple Chronic Conditions using Multiple Medications. Archives of Gerontology and Geriatrics. 2021. **93**: p. 104326 DOI: <https://doi.org/10.1016/j.archger.2020.104326>.

5. Kumar A, et al. Examining the Association Between Comorbidity Indexes and Functional Status in Hospitalized Medicare Fee-for-Service Beneficiaries. Phys Ther. 2016. **96**(2): p. 232-40 DOI: <https://doi.org/10.2522/ptj.20150039>.

6. Lekan DA, et al. Comparison of a Frailty Risk Score and Comorbidity Indices for Hospital Readmission Using Electronic Health Record Data. Res Gerontol Nurs. 2021. **14**(2): p. 91-103 DOI: <https://doi.org/10.3928/19404921-20210115-03>.

7. Mehta HB, et al. Development and Validation of the Summary Elixhauser Comorbidity Score for Use With ICD-10-CM-Coded Data Among Older Adults. Ann Intern Med. 2022. **175**(10): p. 1423-1430 DOI: <https://doi.org/10.7326/m21-4204>.

8. Rosen CB, et al. Multimorbidity Confers Greater Risk for Older Patients in Emergency General Surgery Than the Presence of Multiple Comorbidities: A Retrospective Observational Study. Med Care. 2022. **60**(8): p. 616-622 DOI: <https://doi.org/10.1097/mlr.0000000000001733>.

9. Rosen CB, et al. Analyzing Impact of Multimorbidity on Long-Term Outcomes after Emergency General Surgery: A Retrospective Observational Cohort Study. J Am Coll Surg. 2022. **235**(5): p. 724-735 DOI: <https://doi.org/10.1097/xcs.0000000000000303>.

10. Sangha O, et al. The Self-Administered Comorbidity Questionnaire: a new method to assess comorbidity for clinical and health services research. Arthritis Rheum. 2003. **49**(2): p. 156-63 DOI: <https://doi.org/10.1002/art.10993>.

11. Schear S, et al. Multimorbidity and Opioid Prescribing in Hospitalized Older Adults. J Palliat Med. 2020. **23**(4): p. 475-482 DOI: <https://doi.org/10.1089/jpm.2019.0260>.

12. Shih SL, et al. Functional Status Outperforms Comorbidities as a Predictor of 30-Day Acute Care Readmissions in the Inpatient Rehabilitation Population. J Am Med Dir Assoc. 2016. **17**(10): p. 921-6 DOI: <https://doi.org/10.1016/j.jamda.2016.06.003>.

13. Shih SL, et al. Functional Status Outperforms Comorbidities in Predicting Acute Care Readmissions in Medically Complex Patients. J Gen Intern Med. 2015. **30**(11): p. 1688-95 DOI: <https://doi.org/10.1007/s11606-015-3350-2>.

14. Silber JH, et al. The Safety of Performing Surgery at Ambulatory Surgery Centers Versus Hospital Outpatient Departments in Older Patients With or Without Multimorbidity. Med Care. 2023. **61**(5): p. 328-337 DOI: <https://doi.org/10.1097/mlr.0000000000001836>.

15. Wei MY and Cho J. Readmissions and postdischarge mortality by race and ethnicity among Medicare beneficiaries with multimorbidity. J Am Geriatr Soc. 2023. **71**(6): p. 1749-1758 DOI: <https://doi.org/10.1111/jgs.18251>.

16. Wei MY. Multimorbidity, 30-Day Readmissions, and Postdischarge Mortality Among Medicare Beneficiaries Using a New ICD-Coded Multimorbidity-Weighted Index. J Gerontol A Biol Sci Med Sci. 2023. **78**(4): p. 727-734 DOI: <https://doi.org/10.1093/gerona/glac242>.

17. Chan TC, et al. Validation study of Charlson Comorbidity Index in predicting mortality in Chinese older adults. Geriatr Gerontol Int. 2014. **14**(2): p. 452-7 DOI: <https://doi.org/10.1111/ggi.12129>.

18. Chen H, Chen Y, and Cui B. The association of multimorbidity with healthcare expenditure among the elderly patients in Beijing, China. Arch Gerontol Geriatr. 2018. **79**: p. 32-38 DOI: <https://doi.org/10.1016/j.archger.2018.07.008>.

19. Cheng C, Inder K, and Chan SW. The relationship between coping strategies and psychological distress in Chinese older adults with multiple chronic conditions. Australas J Ageing. 2021. **40**(4): p. 397-405 DOI: <https://doi.org/10.1111/ajag.12946>.

20. Chow SK and Wong FK. A randomized controlled trial of a nurse-led case management programme for hospital-discharged older adults with co-morbidities. J Adv Nurs. 2014. **70**(10): p. 2257-71 DOI: <https://doi.org/10.1111/jan.12375>.

21. Lv J, et al. Research on the frailty status and adverse outcomes of elderly patients with multimorbidity. BMC Geriatr. 2022. **22**(1): p. 560 DOI: <https://doi.org/10.1186/s12877-022-03194-1>.

22. Zhang Z, Yang H, and Luo M. Association Between Charlson Comorbidity Index and Community-Acquired Pressure Injury in Older Acute Inpatients in a Chinese Tertiary Hospital. Clin Interv Aging. 2021. **16**: p. 1987-1995 DOI: <https://doi.org/10.2147/cia.s338967>.

23. Zhang XM, et al. Effect of the Age-Adjusted Charlson Comorbidity Index on All-Cause Mortality and Readmission in Older Surgical Patients: A National Multicenter, Prospective Cohort Study. Front Med (Lausanne). 2022. **9**: p. 896451 DOI: <https://doi.org/10.3389/fmed.2022.896451>.

24. Zhang L, et al. A Multicenter Study of Multimorbidity in Older Adult Inpatients in China. J Nutr Health Aging. 2020. **24**(3): p. 269-276 DOI: <https://doi.org/10.1007/s12603-020-1311-x>.

25. Canevelli M, et al. Counting deficits or diseases? The agreement between frailty and multimorbidity in subjects with cognitive disturbances. Aging Clin Exp Res. 2020. **32**(1): p. 179-182 DOI: <https://doi.org/10.1007/s40520-019-01161-2>.

26. Corrao S, et al. Sex-Differences in the Pattern of Comorbidities, Functional Independence, and Mortality in Elderly Inpatients: Evidence from the RePoSI Register. J Clin Med. 2019. **8**(1) DOI: <https://doi.org/10.3390/jcm8010081>.

27. Corrao S, et al. Comorbidity does not mean clinical complexity: evidence from the RePoSI register. Intern Emerg Med. 2020. **15**(4): p. 621-628 DOI: <https://doi.org/10.1007/s11739-019-02211-3>.

28. Guido D, et al. A comorbidity prognostic effect on post-hospitalization outcome in a geriatric rehabilitation setting: the pivotal role of functionality, assessed by mediation model, and association with the Brass index. Aging Clin Exp Res. 2015. **27**(6): p. 849-56 DOI: <https://doi.org/10.1007/s40520-015-0360-1>.

29. Incalzi RA, et al. The interaction between age and comorbidity contributes to predicting the mortality of geriatric patients in the acute-care hospital. J Intern Med. 1997. **242**(4): p. 291-8 DOI: <https://doi.org/10.1046/j.1365-2796.1997.00132.x>.

30. Novella A, et al. Comparison between drug therapy-based comorbidity indices and the Charlson Comorbidity Index for the detection of severe multimorbidity in older subjects. Aging Clin Exp Res. 2021. **33**(7): p. 1929-1935 DOI: <https://doi.org/10.1007/s40520-020-01706-w>.

31. Novella A, et al. Relation between drug therapy-based comorbidity indices, Charlson's comorbidity index, polypharmacy and mortality in three samples of older adults. Arch Gerontol Geriatr. 2022. **100**: p. 104649 DOI: <https://doi.org/10.1016/j.archger.2022.104649>.

32. Rozzini R, et al. Geriatric Index of Comorbidity: validation and comparison with other measures of comorbidity. Age Ageing. 2002. **31**(4): p. 277-85 DOI: <https://doi.org/10.1093/ageing/31.4.277>.

33. Amasene M, et al. Malnutrition and Poor Physical Function Are Associated With Higher Comorbidity Index in Hospitalized Older Adults. Front Nutr. 2022. **9**: p. 920485 DOI: <https://doi.org/10.3389/fnut.2022.920485>.

34. Briongos-Figuero LS, et al. Evaluation and characterization of multimorbidity profiles, resource consumption and healthcare needs in extremely elderly people. Int J Qual Health Care. 2020. **32**(4): p. 266-270 DOI: <https://doi.org/10.1093/intqhc/mzaa022>.

35. Clerencia-Sierra M, et al. Multimorbidity Patterns in Hospitalized Older Patients: Associations among Chronic Diseases and Geriatric Syndromes. PLoS One. 2015. **10**(7): p. e0132909 DOI: <https://doi.org/10.1371/journal.pone.0132909>.

36. Díez-Manglano J, et al. Excessive polypharmacy and survival in polypathological patients. Eur J Clin Pharmacol. 2015. **71**(6): p. 733-739 DOI: <https://doi.org/10.1007/s00228-015-1837-8>.

37. Enríquez-Gómez A, et al. Comparison of a polypharmacy-based scale with Charlson comorbidity index to predict 6-month mortality in chronic complex patients after an ED visit. Br J Clin Pharmacol. 2022. **88**(4): p. 1795-1803 DOI: <https://doi.org/10.1111/bcp.15096>.

38. Martínez-Velilla N, Cambra-Contin K, and Ibáñez-Beroiz B. Comorbidity and prognostic indices do not improve the 5-year mortality prediction of components of comprehensive geriatric assessment in hospitalized older patients. BMC Geriatr. 2014. **14**: p. 64 DOI: <https://doi.org/10.1186/1471-2318-14-64>.

39. Oterino-Moreira I, et al. Comparison of Three Comorbidity Measures for Predicting In-Hospital Death through a Clinical Administrative Nacional Database. Int J Environ Res Public Health. 2022. **19**(18) DOI: <https://doi.org/10.3390/ijerph191811262>.

40. Aubert CE, et al. Multimorbidity and healthcare resource utilization in Switzerland: a multicentre cohort study. BMC Health Services Research. 2019. **19**(1): p. 708 DOI: <https://doi.org/10.1186/s12913-019-4575-2>.

41. Kutz A, et al. Association of Interprofessional Discharge Planning Using an Electronic Health Record Tool With Hospital Length of Stay Among Patients with Multimorbidity: A Nonrandomized Controlled Trial. JAMA Netw Open. 2022. **5**(9): p. e2233667 DOI: <https://doi.org/10.1001/jamanetworkopen.2022.33667>.

42. Müller M, et al. Association of in-hospital multimorbidity with healthcare outcomes in Swiss medical inpatients. Swiss Med Wkly. 2021. **151**: p. w20405 DOI: <https://doi.org/10.4414/smw.2021.20405>.

43. Schneider C, et al. Comparison of 6 Mortality Risk Scores for Prediction of 1-Year Mortality Risk in Older Adults With Multimorbidity. JAMA Netw Open. 2022. **5**(7): p. e2223911 DOI: <https://doi.org/10.1001/jamanetworkopen.2022.23911>.

44. Zekry D, et al. Geriatrics index of comorbidity was the most accurate predictor of death in geriatric hospital among six comorbidity scores. J Clin Epidemiol. 2010. **63**(9): p. 1036-44 DOI: <https://doi.org/10.1016/j.jclinepi.2009.11.013>.

45. Zekry D, et al. Prospective comparison of 6 comorbidity indices as predictors of 1-year post-hospital discharge institutionalization, readmission, and mortality in elderly individuals. J Am Med Dir Assoc. 2012. **13**(3): p. 272-8 DOI: <https://doi.org/10.1016/j.jamda.2010.11.011>.

46. Bahat G, et al. Assessments of functional status, comorbidities, polypharmacy, nutritional status and sarcopenia in Turkish community-dwelling male elderly. Aging Male. 2013. **16**(2): p. 67-72 DOI: <https://doi.org/10.3109/13685538.2013.771329>.

47. Bahat G, et al. Comorbidities, polypharmacy, functionality and nutritional status in Turkish community-dwelling female elderly. Aging Clin Exp Res. 2014. **26**(3): p. 255-9 DOI: <https://doi.org/10.1007/s40520-014-0229-8>.

48. Canaslan K, et al. Predictivity of the comorbidity indices for geriatric syndromes. BMC Geriatr. 2022. **22**(1): p. 440 DOI: <https://doi.org/10.1186/s12877-022-03066-8>.

49. Das M, et al. Prediction of mortality with Charlson Comorbidity Index in super-elderly patients admitted to a tertiary referral hospital. Çukurova Araştırmaları Dergisi. 2022. **47**: p. 199-207 DOI: <https://doi.org/10.17826/cumj.1017164>.

50. Ünlü EH, et al. Comparison of the Modified 5-item Frailty Index with the American Society of Anaesthesiologists Classification and Charlson Age Comorbidity Index for predicting postoperative outcomes in geriatric patients: A Prospective Observational Study. Turkish Journal of Geriatrics. 2022. **25**(4) DOI: <https://doi.org/10.31086/tjgeri.2022.320>.

51. Bernard S, Inderjeeth C, and Raymond W. Higher Charlson Comorbidity Index scores do not influence Functional Independence Measure score gains in older rehabilitation patients. Australas J Ageing. 2016. **35**(4): p. 236-241 DOI: <https://doi.org/10.1111/ajag.12351>.

52. Chan CH, et al. The association of comorbidity measures and mortality in geriatric rehabilitation inpatients by cancer status: RESORT. Support Care Cancer. 2021. **29**(8): p. 4513-4519 DOI: <https://doi.org/10.1007/s00520-020-05967-z>.

53. Shakib S, et al. Effect of a Multidisciplinary Outpatient Model of Care on Health Outcomes in Older Patients with Multimorbidity: A Retrospective Case Control Study. PLoS One. 2016. **11**(8): p. e0161382 DOI: <https://doi.org/10.1371/journal.pone.0161382>.

54. Williams A, et al. Effect of age and comorbidity on the ability of quick-Sequential Organ Failure Assessment score to predict outcome in emergency department patients with suspected infection. Emerg Med Australas. 2021. **33**(4): p. 679-684 DOI: <https://doi.org/10.1111/1742-6723.13703>.

55. Chou HC, Huang CT, and Sheng WH. Differential roles of comorbidity burden and functional status in elderly and non-elderly patients with infections in general wards. J Formos Med Assoc. 2020. **119**(4): p. 821-828 DOI: <https://doi.org/10.1016/j.jfma.2019.08.032>.

56. Lai HY, et al. Development of frailty index using ICD-10 codes to predict mortality and rehospitalization of older adults: An update of the multimorbidity frailty index. Arch Gerontol Geriatr. 2022. **100**: p. 104646 DOI: <https://doi.org/10.1016/j.archger.2022.104646>.

57. Liang HY, et al. Effectiveness of a Nurse-Led Tele-Homecare Program for Patients With Multiple Chronic Illnesses and a High Risk for Readmission: A Randomized Controlled Trial. J Nurs Scholarsh. 2021. **53**(2): p. 161-170 DOI: <https://doi.org/10.1111/jnu.12622>.

58. Peng LN, et al. Nutritional status plays the mediating role of the functional status and comorbidity among older patients admitted to the Geriatric Evaluation and Management Unit: A Tobit model application. European Geriatric Medicine. 2014. **5**(2): p. 87-91 DOI: <https://doi.org/10.1016/j.eurger.2013.12.003>.

59. Patrick L, et al. Medical comorbidity and rehabilitation efficiency in geriatric inpatients. J Am Geriatr Soc. 2001. **49**(11): p. 1471-7 DOI: <https://doi.org/10.1046/j.1532-5415.2001.4911239.x>.

60. Susser SR, McCusker J, and Belzile E. Comorbidity information in older patients at an emergency visit: self-report vs. administrative data had poor agreement but similar predictive validity. J Clin Epidemiol. 2008. **61**(5): p. 511-5 DOI: <https://doi.org/10.1016/j.jclinepi.2007.07.009>.

61. Buurman BM, et al. Acute and chronic diseases as part of multimorbidity in acutely hospitalized older patients. Eur J Intern Med. 2016. **27**: p. 68-75 DOI: <https://doi.org/10.1016/j.ejim.2015.09.021>.

62. Dias A, et al. Comorbidity burden assessment in older people admitted to a Portuguese University Hospital. Aging Clin Exp Res. 2015. **27**(3): p. 323-8 DOI: <https://doi.org/10.1007/s40520-014-0280-5>.

63. Félix IB and Henriques A. Medication adherence and related determinants in older people with multimorbidity: A cross-sectional study. Nurs Forum. 2021. **56**(4): p. 834-843 DOI: <https://doi.org/10.1111/nuf.12619>.

64. Chua MT, et al. Comparing Comorbidity Polypharmacy Score and Charlson Comorbidity Index in predicting outcomes in older trauma patients. Injury. 2023. **54**(4): p. 1113-1118 DOI: <https://doi.org/10.1016/j.injury.2023.02.031>.

65. Tan C, et al. Disability impacts length of stay in general internal medicine patients. J Gen Intern Med. 2014. **29**(6): p. 885-90 DOI: <https://doi.org/10.1007/s11606-014-2815-z>.

66. Sara HH, Chowdhury MAB, and Haque MA. Multimorbidity among elderly in Bangladesh. Aging Med (Milton). 2018. **1**(3): p. 267-275 DOI: <https://doi.org/10.1002/agm2.12047>.

67. Juul-Larsen HG, et al. Differences in function and recovery profiles between patterns of multimorbidity among older medical patients the first year after an acute admission-An exploratory latent class analysis. Arch Gerontol Geriatr. 2020. **86**: p. 103956 DOI: <https://doi.org/10.1016/j.archger.2019.103956>.

68. Bahrmann A, et al. The Charlson Comorbidity and Barthel Index predict length of hospital stay, mortality, cardiovascular mortality and rehospitalization in unselected older patients admitted to the emergency department. Aging Clin Exp Res. 2019. **31**(9): p. 1233-1242 DOI: <https://doi.org/10.1007/s40520-018-1067-x>.

69. Beloosesky Y, Weiss A, and Mansur N. Validity of the Medication-based Disease Burden Index compared with the Charlson Comorbidity Index and the Cumulative Illness Rating Scale for geriatrics: a cohort study. Drugs Aging. 2011. **28**(12): p. 1007-14 DOI: <https://doi.org/10.2165/11597040-000000000-00000>.

70. Helvik AS, Engedal K, and Selbæk G. Three-year mortality in previously hospitalized older patients from rural areas--the importance of co-morbidity and self-reported poor health. BMC Geriatr. 2013. **13**: p. 17 DOI: <https://doi.org/10.1186/1471-2318-13-17>.

71. Capisizu A, et al. Findings regarding the relationships between sociodemographic, psychological, comorbidity factors, and functional status, in geriatric inpatients. Adv Exp Med Biol. 2015. **821**: p. 45-55 DOI: <https://doi.org/10.1007/978-3-319-08939-3_9>.

72. Olsson T, Terent A, and Lind L. Charlson Comorbidity Index can add prognostic information to Rapid Emergency Medicine Score as a predictor of long-term mortality. Eur J Emerg Med. 2005. **12**(5): p. 220-4 DOI: <https://doi.org/10.1097/00063110-200510000-00004>.

73. Thanakiattiwibun C, et al. Multimorbidity, healthcare utilization, and quality of life for older patients undergoing surgery: A prospective study. Medicine (Baltimore). 2023. **102**(13): p. e33389 DOI: <https://doi.org/10.1097/md.0000000000033389>.

74. Kabboord AD, et al. The modified functional comorbidity index performed better than the Charlson index and original functional comorbidity index in predicting functional outcome in geriatric rehabilitation: a prospective observational study. BMC Geriatr. 2020. **20**(1): p. 114 DOI: <https://doi.org/10.1186/s12877-020-1498-z>.

75. Vu HM, et al. Effects of Chronic Comorbidities on the Health-Related Quality of Life among Older Patients after Falls in Vietnamese Hospitals. Int J Environ Res Public Health. 2019. **16**(19) DOI: <https://doi.org/10.3390/ijerph16193623>.
